# Effects of Foliar-Applied PAA@Mn_3_O_4_ Nanoparticles, Methyl Jasmonate, and γ-Aminobutyric Acid on Growth and Yield Performance of Sugar Beet (*Beta vulgaris* L.) Under Salt-Affected Conditions

**DOI:** 10.3390/plants15172729

**Published:** 2026-09-07

**Authors:** Zijian Zhang, Guansen Cao, Lihua Yang, Yaqing Sun, Guolong Li, Ningning Li

**Affiliations:** College of Agronomy, Inner Mongolia Agricultural University, Hohhot 010019, China; zacharygrant@emails.imau.edu.cn (Z.Z.); wojiaocgs2021@163.com (G.C.); 18313593379@163.com (L.Y.); syaqing@imau.edu.cn (Y.S.); lglg@imau.edu.cn (G.L.)

**Keywords:** γ-aminobutyric acid (GABA), methyl jasmonate (MeJA), PAA@Mn_3_O_4_ nanoparticles (PMO), salt stress, sugar beet

## Abstract

Soil salinity restricts sugar beet growth and productivity, creating a need for effective agronomic approaches to improve plant performance under salt-affected conditions. However, the comparative responses of sugar beet to different foliar-applied exogenous substances under controlled NaCl stress and field saline–alkali conditions remain insufficiently understood. In this study, sugar beet (*Beta vulgaris* L.) cultivar ‘HI0479’ was used to evaluate the effects of different concentrations of PAA@Mn_3_O_4_ nanoparticles (PMO), methyl jasmonate (MeJA), and γ-aminobutyric acid (GABA). A pot experiment was first conducted under controlled NaCl stress to identify concentrations for subsequent field evaluation, followed by a one-season field experiment under non-saline–alkali and saline–alkali soil conditions. Based on the overall responses of growth and physiological traits, 100 mg L^−1^ PMO, 100 mg L^−1^ MeJA, and 1000 mg L^−1^ GABA were selected for field evaluation. Under the imposed NaCl treatment, these foliar treatments improved growth and physiological performance and were associated with changes in photosynthetic characteristics, antioxidant enzyme activities, MDA content, osmolyte accumulation, and selected growth-related hormone levels. Under saline–alkali field conditions, PMO, MeJA, and GABA increased storage-root yield by 13.59%, 12.69%, and 12.37%, respectively, compared with the control. Although estimated storage-root sugar concentration decreased, the corresponding estimated sugar yields increased by 10.77%, 9.79%, and 7.41%, respectively. These results show that the three foliar treatments produced favorable growth- and yield-related responses under the experimental conditions evaluated while also revealing a trade-off between storage-root yield and estimated storage-root sugar concentration. The findings provide a basis for further agronomic evaluation of foliar PMO, MeJA, and GABA in sugar beet grown under salt-affected conditions.

## 1. Introduction

Soil salinization is a major form of land degradation that constrains agricultural productivity and sustainable crop production worldwide [1]. China has extensive saline–alkali land resources, including large areas with potential for agricultural utilization [2]. The Hetao Irrigation District in Inner Mongolia is an important agricultural production region in northern China, where soil salinization remains a major constraint on crop productivity [3]. Sugar beet (*Beta vulgaris* L.) is an economically important sugar crop with moderate tolerance to salt stress and is widely cultivated in northern China [4]. Nevertheless, excessive salt stress can inhibit seedling growth and root development and disturb photosynthesis, redox balance, osmotic regulation, and growth-related physiological processes, ultimately reducing crop productivity [5]. Therefore, developing practical agronomic approaches to improve sugar beet performance under salt-affected conditions is of considerable importance [6].

Foliar application of exogenous substances has been widely investigated as an approach to regulate plant responses to abiotic stress [7,8]. Among these substances, PAA@Mn_3_O_4_ nanoparticles (PMO), methyl jasmonate (MeJA), and γ-aminobutyric acid (GABA) have shown potential to modify plant physiological responses under adverse conditions [9,10,11]. As a manganese-based functional nanomaterial, PAA@Mn_3_O_4_ nanoparticles consist of a Mn_3_O_4_ core and a polyacrylic acid (PAA) surface coating layer. Previous studies have characterized the physicochemical properties of this nanomaterial system and reported its nanoscale structure, stable surface characteristics, and redox-related activity associated with manganese oxides [12]. In vitro studies have further shown that PAA@Mn_3_O_4_ nanoparticles exhibit ROS-related activity, and previous studies have reported changes in antioxidant-related physiological responses in plants following their application under stress conditions [12,13]. Therefore, PAA@Mn_3_O_4_ represents a novel exogenous material for exploring the potential involvement of nanomaterials in the regulation of plant stress responses. Methyl jasmonate (MeJA) is an important jasmonate-related signaling molecule involved in the regulation of plant growth, development, and stress responses [14]. In crops such as turnip (*Brassica rapa* L.) and walnut (*Juglans regia* L.), exogenous MeJA has been reported to modulate genes associated with phenylpropanoid biosynthesis, glutathione metabolism, and hormone signaling pathways, accompanied by increased antioxidant enzyme activities and enhanced osmolyte accumulation [15]. These coordinated responses may contribute to regulation of ROS-related processes and reduction in oxidative damage, thereby improving plant growth and physiological performance under adverse conditions [16]. γ-Aminobutyric acid (GABA) is a four-carbon non-protein amino acid that is widely distributed in plants and participates in multiple biological processes [17]. Under salt stress conditions, endogenous GABA levels commonly undergo dynamic changes. Previous studies have reported that exogenous GABA can influence osmolyte accumulation, antioxidant systems, and metabolic processes, thereby affecting plant physiological performance under adverse environmental conditions. Studies in crops such as maize (*Zea mays* L.) and wheat (*Triticum aestivum* L.) have further shown that GABA application can regulate photosynthetic characteristics and antioxidant-related physiological processes [11,18].

Because PMO, MeJA, and GABA represent different types of exogenous regulatory approaches, including nanomaterials, signaling molecules, and metabolic regulators, respectively, comparing these substances within the same experimental framework may provide insights into how different regulatory strategies influence salt-stress responses and field performance of sugar beet. Therefore, this study selected PMO, MeJA, and GABA as representative exogenous substances and systematically evaluated their effects on sugar beet growth, physiological characteristics, and yield formation under controlled NaCl treatment and saline–alkali field conditions.

Previous studies on exogenous regulation of salt-stressed sugar beet have mainly focused on conventional substances such as salicylic acid, brassinosteroids, and melatonin [19], whereas systematic concentration screening followed by field evaluation remains limited. In particular, the responses of sugar beet to PMO, MeJA, and GABA have not been comparatively evaluated within the same experimental framework. We therefore hypothesized that foliar application of these three exogenous substances at selected concentrations could differentially affect the growth and physiological performance of sugar beet subjected to controlled NaCl treatment and influence agronomic performance under field saline–alkali conditions. To test this hypothesis, we first conducted a pot experiment to identify concentrations of PMO, MeJA, and GABA for subsequent field evaluation and assessed their effects on seedling growth, photosynthetic characteristics, antioxidant enzyme activities, MDA content, osmolyte accumulation, and selected growth-related hormone levels. The selected concentrations were subsequently evaluated in a one-season field experiment under non-saline–alkali and saline–alkali soil conditions to assess their effects on plant growth, physiological traits, storage-root yield, estimated storage-root sugar concentration, and estimated sugar yield. The study was intended to provide a comparative basis for understanding the physiological responses of sugar beet to these foliar treatments and to support their further agronomic evaluation under salt-affected production conditions.

**Figure 1:**
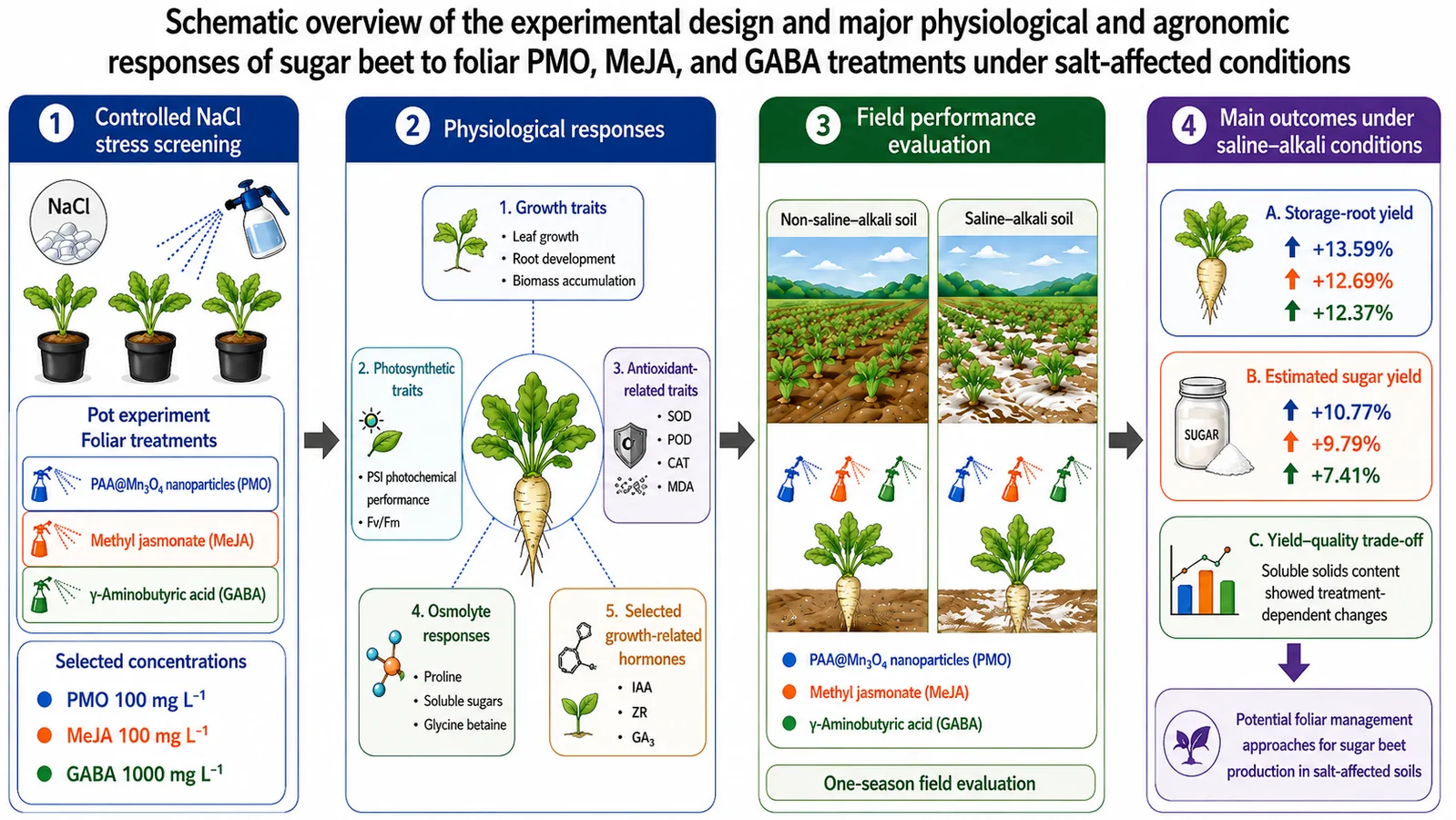
Schematic overview of experimental design and major physiological and agronomic responses of sugar beet to foliar PMO, MeJA, and GABA treatments under salt-affected conditions.

A schematic model summarizing the experimental design and the major physiological and agronomic responses induced by foliar PMO, MeJA, and GABA treatments under salt-affected conditions is presented in Figure 1.

## 2. Results

### 2.1. Effects of Three Exogenous Substances on the Growth Phenotype of Sugar Beet Seedlings Under Salt Stress

**Figure 2:**
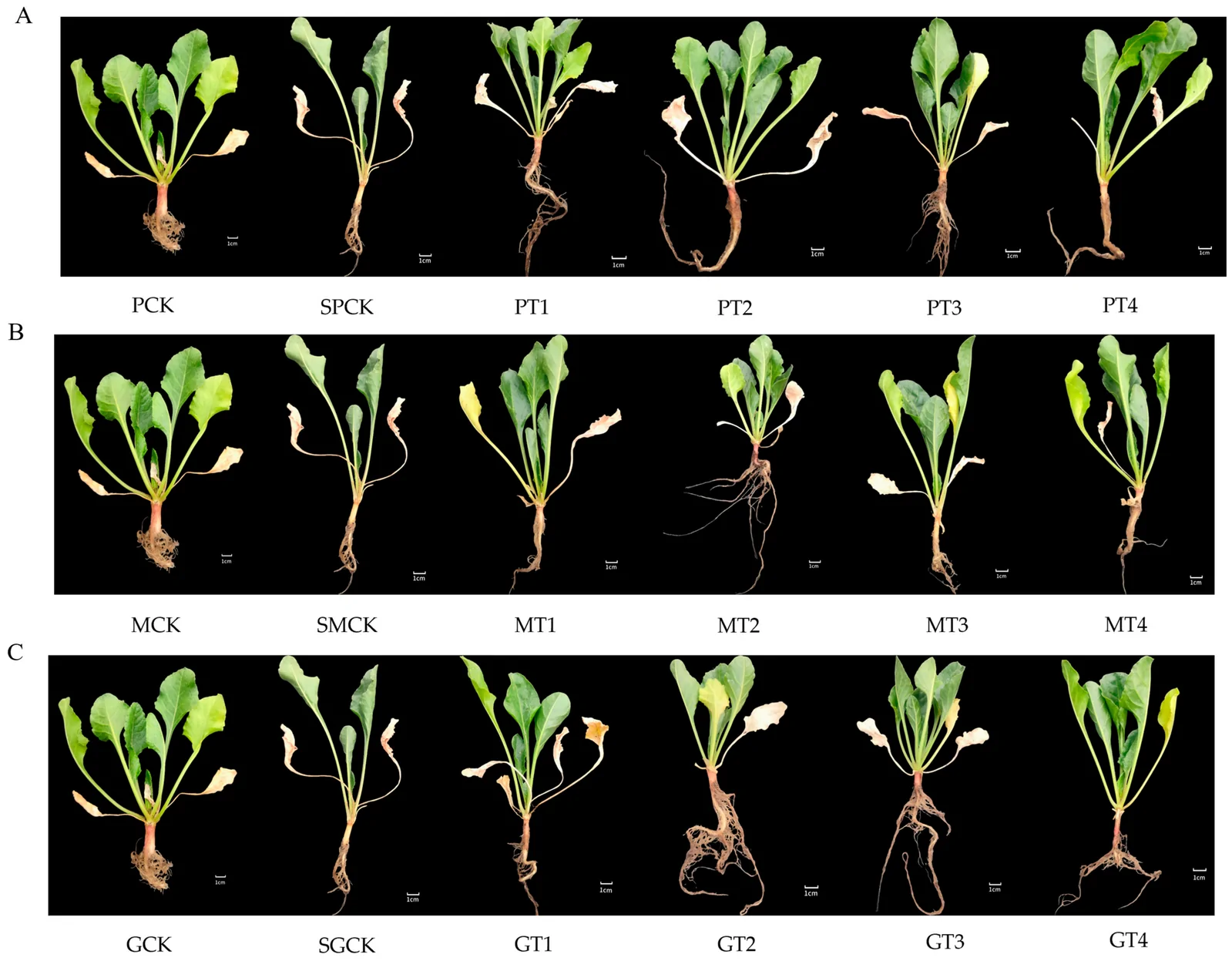
Effects of Three Exogenous Substances on the Growth Phenotype of Sugar Beet Seedlings under Salt Stress. (**A**–**C**) Representative whole-plant phenotypes of sugar beet seedlings treated with PAA@Mn_3_O_4_ nanoparticles (PMO), methyl jasmonate (MeJA), and γ-aminobutyric acid (GABA), respectively. PCK, MCK, and GCK denote the same shared non-NaCl-stressed control (CK), whereas SPCK, SMCK, and SGCK denote the same shared NaCl-stressed control (SCK); these labels are repeated only to facilitate comparison within each treatment series and do not represent independent control groups. PT1–PT4, MT1–MT4, and GT1–GT4 represent the four concentration treatments of PMO, MeJA, and GABA, respectively.

NaCl treatment markedly altered the growth phenotype of sugar beet seedlings, while foliar application of PMO, MeJA, and GABA was associated with partial improvements in seedling growth characteristics. The observed responses varied among the three exogenous substances and across the tested concentrations (Figure 2A–C). Under non-NaCl-stressed conditions, the shared control seedlings, represented by the labels PCK, MCK, and GCK for the three treatment series, showed vigorous growth: shoots bore expanded, dark green leaves with large leaf area, and the root system was well developed with robust taproot elongation and dense fibrous roots. Under NaCl treatment, the shared salt-stressed control seedlings, represented by SPCK, SMCK, and SGCK, showed pronounced growth inhibition. Leaves displayed wilting and curling, with progressive chlorosis and necrosis occurring at the leaf tips and margins. Root development was particularly impaired: taproot thickening was suppressed, and the number of fibrous roots decreased significantly (Figure 2A–C).

**Figure 3:**
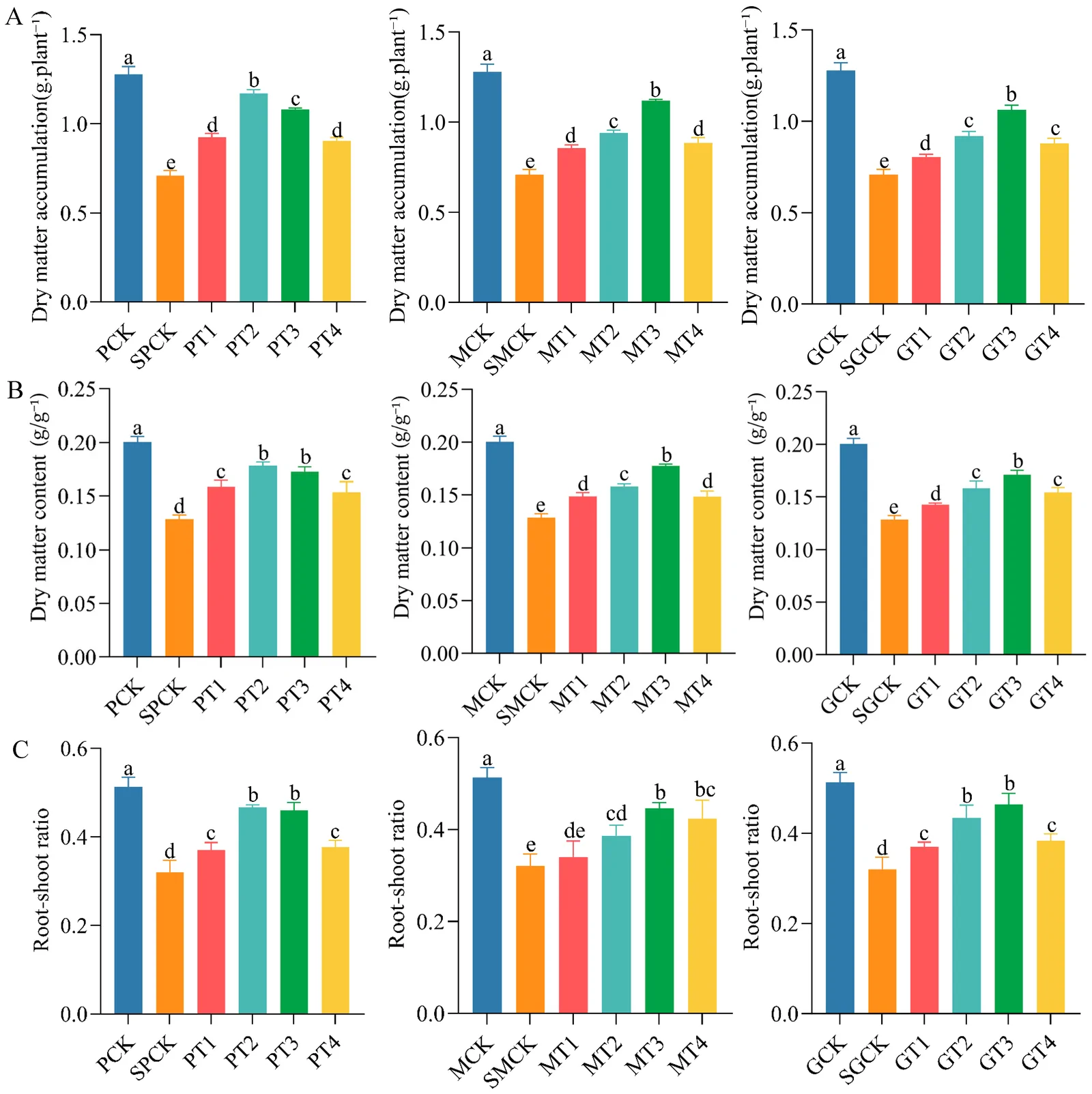
Effects of Three Foliar Treatments on Root Architecture of Sugar Beet Seedlings under NaCl Stress. (**A**) Dry matter accumulation per plant; (**B**) dry matter content; (**C**) root–shoot ratio. PCK, MCK, and GCK denote the same shared non-NaCl-stressed control (CK), whereas SPCK, SMCK, and SGCK denote the same shared NaCl-stressed control (SCK); these labels are repeated only to facilitate comparison within each treatment series and do not represent independent control groups. PT1–PT4, MT1–MT4, and GT1–GT4 represent four concentration treatments of PMO, MeJA, and GABA, respectively. Data are presented as mean ± SD from three independent experimental replicates (*n* = 3). Different lowercase letters indicate significant differences among treatments within the corresponding treatment series according to Duncan’s multiple range test (*p* < 0.05).

Foliar application of the three exogenous substances produced varying improvements in the measured growth traits. Morphological observations combined with quantitative measurements revealed that salt stress significantly reduced leaf number and taproot diameter of sugar beet seedlings by 38.70% and 41.10%, respectively, relative to the normal control (*p* < 0.05). Taproot length decreased by 10.00%, but this change was not statistically significant (Figure 3A–C). Among the tested concentrations, PT2 (PMO), MT3 (MeJA), and GT3 (GABA) showed comparatively favorable overall responses across the measured growth traits and were selected for subsequent field evaluation. Compared with their respective salt stress controls, PT2 treatment significantly increased leaf number, taproot length and taproot diameter by 47.40%, 76.90% and 32.10%, respectively; MT3 treatment increased these parameters by 42.10%, 30.70% and 13.80%, respectively; and GT3 treatment increased them by 57.90%, 53.40% and 15.70%, respectively (*p* < 0.05). Plant growth responses to salt stress are essentially an adaptive remodeling process achieved by modulating the synthesis and allocation patterns of photosynthates [20]. As a comprehensive indicator of plant carbon assimilation capacity and overall growth performance, dry matter accumulation is the most straightforward core parameter for assessing the severity of salt stress injury and the efficacy of exogenous regulation. Dry matter content reflects the biosynthetic efficiency and accumulation level of organic matter in plants under adverse conditions, and can indirectly indicate the transport and transformation characteristics of photosynthates. The root–shoot ratio is a key parameter representing the balance of resource allocation between shoots and roots; its dynamic shift represents an important adaptive strategy for plants to optimize water and nutrient uptake under salt stress. To systematically clarify the regulatory effects of three exogenous substances (PMO, MeJA and GABA) on the growth and dry matter partitioning of sugar beet seedlings under salt stress, dry matter accumulation, dry matter content and root–shoot ratio were quantitatively measured in this study. Significant differences among treatments were evaluated using Duncan’s multiple range test (*p* < 0.05).

**Figure 4:**
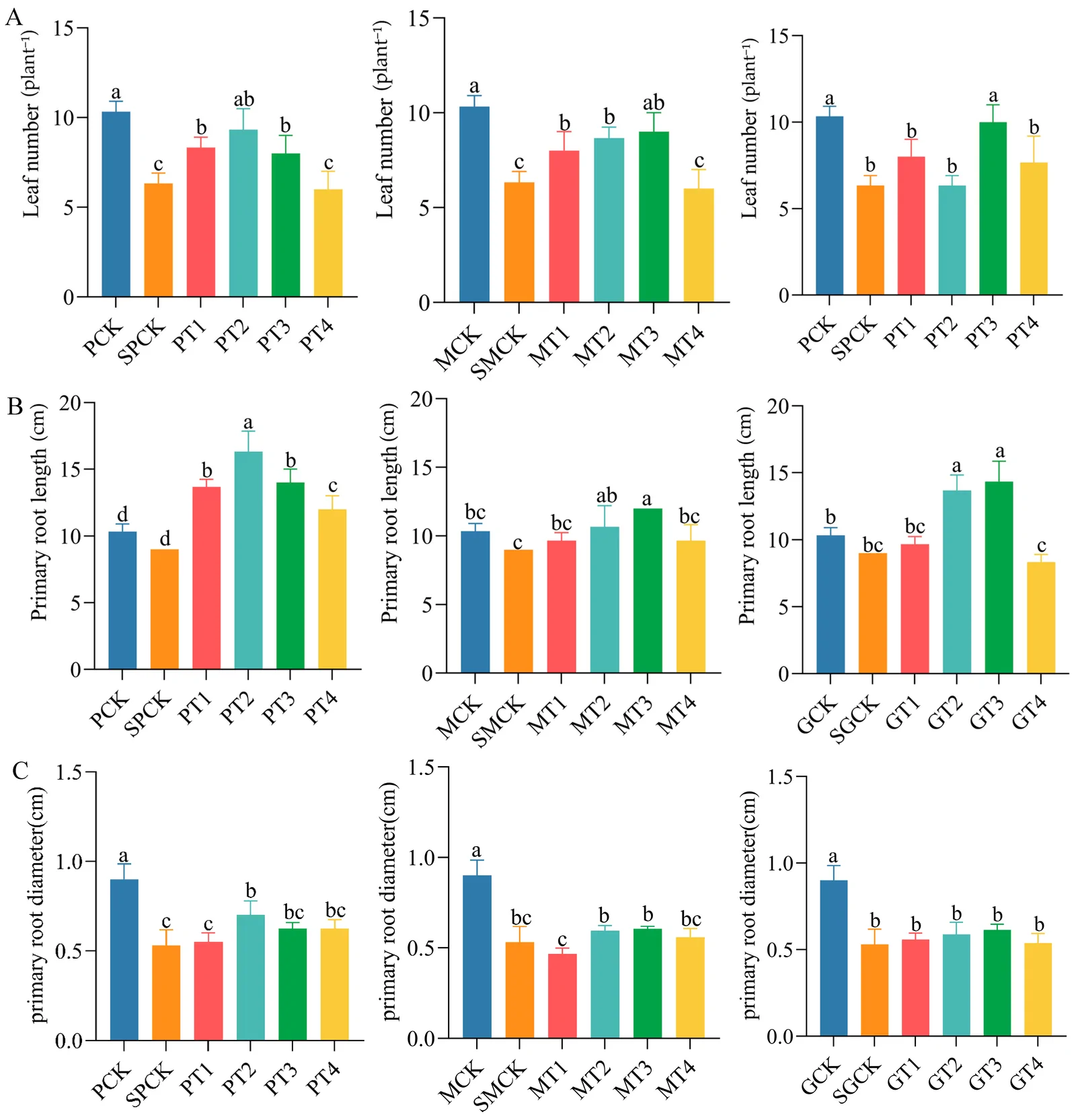
Effects of Foliar PMO, MeJA, and GABA Treatments on Dry Matter Accumulation and Root–Shoot Ratio of Sugar Beet. (**A**) Leaf number; (**B**) taproot length; (**C**) taproot diameter. PCK, MCK, and GCK denote the same shared non-NaCl-stressed control (CK), whereas SPCK, SMCK, and SGCK denote the same shared NaCl-stressed control (SCK); these labels are repeated only to facilitate comparison within each treatment series and do not represent independent control groups. PT1–PT4, MT1–MT4, and GT1–GT4 represent four concentration treatments of PMO, MeJA, and GABA, respectively. Data are presented as mean ± SD from three independent experimental replicates (n = 3). Different lowercase letters indicate significant differences among treatments within the corresponding treatment series according to Duncan’s multiple range test (*p* < 0.05).

The results demonstrated that salt stress significantly suppressed the growth of sugar beet seedlings (Figure 4A–C). Compared with the shared non-NaCl-stressed control (CK), represented by PCK, MCK, and GCK in the three treatment series, the shared NaCl-stressed control (SCK), represented by SPCK, SMCK, and SGCK, showed significant reductions in dry matter accumulation, dry matter content and root–shoot ratio, with decrements of 44.60%, 35.80% and 38.05%, respectively.

Foliar application of all three exogenous substances was associated with varying improvements in dry matter accumulation, dry matter content, and root-to-shoot ratio under NaCl treatment (Figure 4A–C). Among the tested concentrations, PT2 (PMO), MT3 (MeJA), and GT3 (GABA) showed comparatively favorable overall responses across these growth-related traits. Relative to their corresponding salt-stressed controls, PT2 treatment significantly increased dry matter accumulation, dry matter content and root–shoot ratio by 64.80%, 39.10% and 46.90%, respectively; MT3 treatment increased these indices by 28.50%, 38.00% and 40.60%, respectively; and GT3 treatment increased them by 50.30%, 32.90% and 43.80%, respectively. All changes reached statistical significance (*p* < 0.05).

### 2.2. Effects of Three Exogenous Substances on Photosynthetic Characteristics of Sugar Beet Seedlings

**Figure 5:**
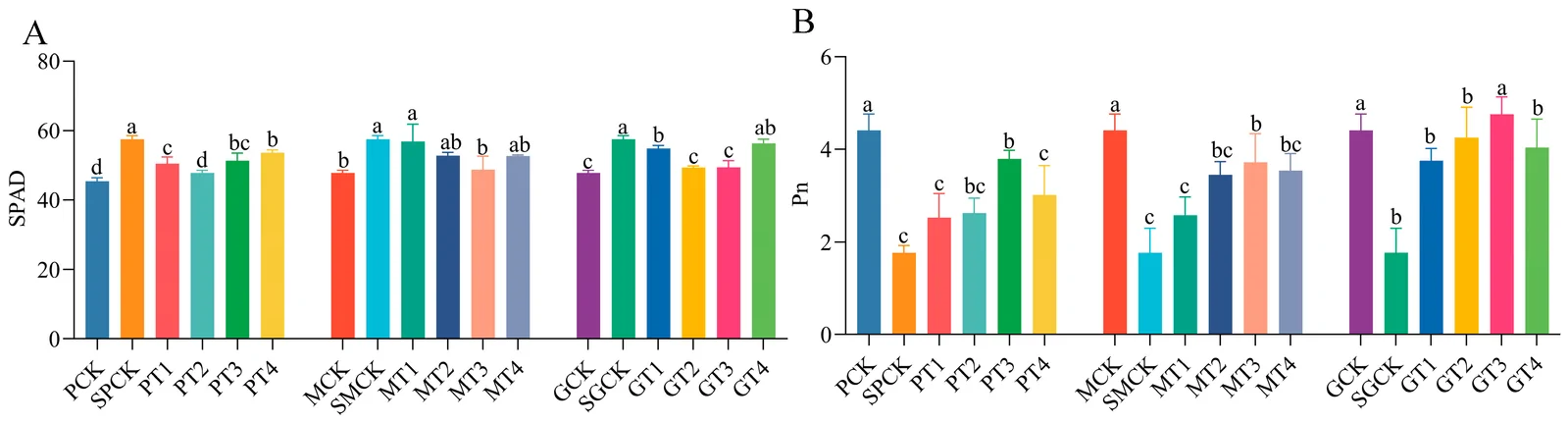
Effects of foliar PMO, MeJA, and GABA treatments on photosynthetic characteristics of sugar beet seedlings under NaCl stress. (**A**) Leaf SPAD value; (**B**) leaf net photosynthetic rate (Pn). Different colors represent the PMO, MeJA, and GABA foliar treatments. PCK, MCK, and GCK denote the same shared non-NaCl-stressed control (CK), whereas SPCK, SMCK, and SGCK denote the same shared NaCl-stressed control (SCK); these labels are repeated only to facilitate comparison within each treatment series and do not represent independent control groups. PT1–PT4, MT1–MT4, and GT1–GT4 represent four concentration treatments of PMO, MeJA, and GABA, respectively. Data are presented as mean ± SD from three independent experimental replicates (n = 3). Different lowercase letters indicate significant differences among treatments within the corresponding treatment series according to Duncan’s multiple range test (*p* < 0.05).

Leaf SPAD value provides a relative index of chlorophyll status and was used to characterize treatment-related changes in sugar beet leaves [21]. Compared with the non-salt-stressed control (Figure 5A), the leaf SPAD value of sugar beet seedlings under salt stress increased significantly by 26.68% (*p* < 0.05). Foliar application of the three exogenous substances—PAA@Mn_3_O_4_ nanoparticles (PMO), methyl jasmonate (MeJA), and γ-aminobutyric acid (GABA)—reduced leaf SPAD values under NaCl treatment. The SPAD values across the three treatment groups exhibited a trend of initial decrease followed by an increase with rising regulator concentrations. Among them, the PT2 (PMO), MT3 (MeJA), GT2 and GT3 (GABA) treatments yielded leaf SPAD values that showed no significant difference from the non-salt-stressed control, and were significantly reduced by 16.90%, 15.25%, 14.19% and 14.35%, respectively, relative to their corresponding salt-stressed controls (*p* < 0.05). These results indicate that foliar application of the three exogenous substances altered leaf SPAD values under the imposed NaCl treatment.

The imposed NaCl treatment significantly reduced the net photosynthetic rate (Pn) of sugar beet seedling leaves. Foliar application of the three exogenous substances increased Pn to varying degrees under the imposed NaCl treatment (Figure 5B), and the net photosynthetic rate of all treatments showed a trend of initial increase followed by a decrease with increasing treatment concentrations. Among the tested concentrations, PT3 (PMO), MT3 (MeJA), and GT3 (GABA) showed the highest Pn values within their respective treatment series, with leaf net photosynthetic rate significantly elevated by 114.12%, 110.17% and 168.36%, respectively, compared with their corresponding salt-stressed controls (*p* < 0.05).

**Figure 6:**
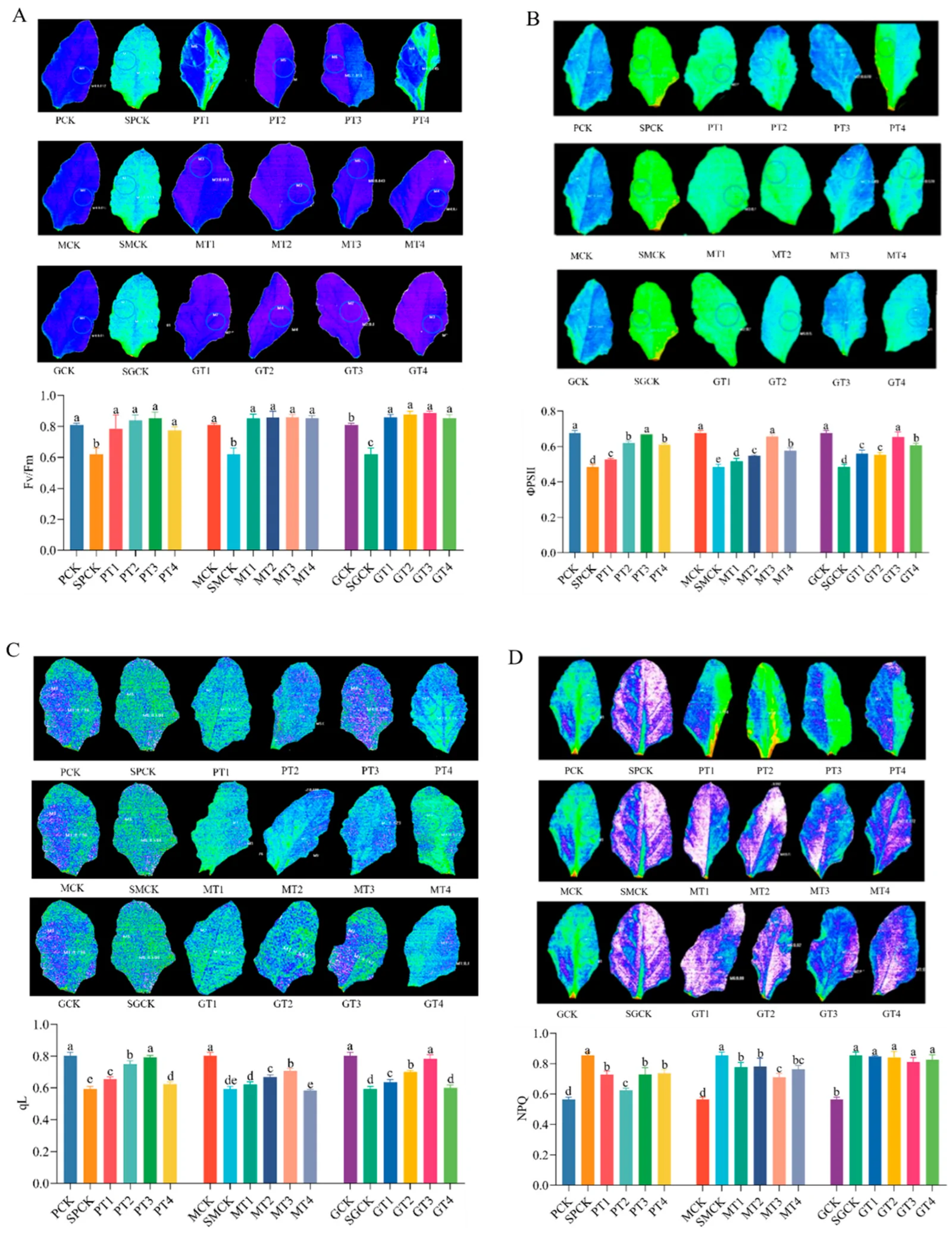
Effects of foliar PMO, MeJA, and GABA treatments on chlorophyll fluorescence characteristics of sugar beet seedlings under NaCl stress. (**A**–**D**) Chlorophyll fluorescence images and quantitative analyses of the maximum photochemical efficiency of PSII (Fv/Fm), effective quantum yield of PSII photochemistry (ΦPSII), photochemical quenching coefficient (qL), and non-photochemical quenching (NPQ), respectively. The treatments were grouped according to the three foliar treatments: PAA@Mn_3_O_4_ nanoparticles (PMO), methyl jasmonate (MeJA), and γ-aminobutyric acid (GABA). PCK, MCK, and GCK denote the same shared non-NaCl-stressed control (CK), whereas SPCK, SMCK, and SGCK denote the same shared NaCl-stressed control (SCK); these labels are repeated only to facilitate comparison within each treatment series and do not represent independent control groups. PT1–PT4, MT1–MT4, and GT1–GT4 represent four concentration treatments of PMO, MeJA, and GABA, respectively. The fluorescence images are presented as representative pseudocolor images, whereas quantitative comparisons are based on the measured fluorescence parameter values. The original numerical color-scale information could not be recovered from the retained image files. Data are presented as mean ± SD from three independent experimental replicates (n = 3). Different lowercase letters indicate significant differences among treatments within the corresponding treatment series according to Duncan’s multiple range test (*p* < 0.05).

Chlorophyll fluorescence parameters provide information on the photochemical performance of PSII under stress conditions [20]. Salt stress significantly altered chlorophyll fluorescence characteristics in sugar beet seedlings. Compared with the corresponding non-NaCl-stressed controls, the maximum photochemical efficiency of PSII (Fv/Fm), effective quantum yield of PSII photochemistry (ΦPSII), and photochemical quenching coefficient (qL) were significantly reduced under NaCl stress, whereas the non-photochemical quenching coefficient (NPQ) increased. Foliar application of PMO, MeJA, and GABA was associated with changes in these fluorescence parameters, although the magnitude of the responses differed among treatments and concentrations (Figure 6).

Representative chlorophyll fluorescence pseudocolor images are shown in Figure 6A–D. Because the original numerical color-scale information could not be recovered from the retained image files, the pseudocolor images were used only for qualitative visualization, whereas treatment comparisons were based on the measured fluorescence parameter values described below.

Quantitative analysis of Fv/Fm (Figure 6A) showed that all concentrations of PMO, MeJA, and GABA significantly increased Fv/Fm compared with the corresponding NaCl-stressed control, whereas no significant differences were observed among different concentrations within the same treatment series. The increases in Fv/Fm were generally consistent across the three foliar treatments, indicating an improvement in the maintenance of PSII maximum photochemical efficiency under NaCl stress.

For ΦPSII (Figure 6B), NaCl stress significantly decreased the effective quantum yield of PSII photochemistry (ΦPSII). All three foliar treatments significantly increased ΦPSII, with the highest values observed for PT3, MT3, and GT3 within the PMO, MeJA, and GABA treatment series, respectively. Compared with the corresponding NaCl-stressed controls, PT3, MT3, and GT3 increased ΦPSII by 39.72%, 36.59%, and 35.69%, respectively.

The variation pattern of qL was similar to that of ΦPSII (Figure 6C). Salt stress significantly reduced qL, whereas PMO, MeJA, and GABA treatments significantly increased qL values. The highest qL values within the respective treatment series were observed for PT3, MT3, and GT3, which increased qL by 34.52%, 19.44%, and 32.77%, respectively, compared with the corresponding NaCl-stressed controls.

In contrast, NPQ showed an opposite response pattern (Figure 6D). NaCl stress significantly increased NPQ, indicating greater non-photochemical energy dissipation under the imposed NaCl treatment. PMO and MeJA treatments significantly reduced NPQ values compared with the NaCl-stressed control, whereas GABA showed no significant overall effect except at the T3 concentration, where NPQ was slightly reduced.

Overall, PMO, MeJA, and GABA treatments were associated with improved PSII-related fluorescence parameters under the imposed NaCl treatment, as reflected by increased Fv/Fm, ΦPSII, and qL and, in some treatments, lower NPQ values.

### 2.3. Effects of Three Exogenous Substances on Antioxidant Enzyme Activities and MDA Content in Sugar Beet Seedlings

**Figure 7:**
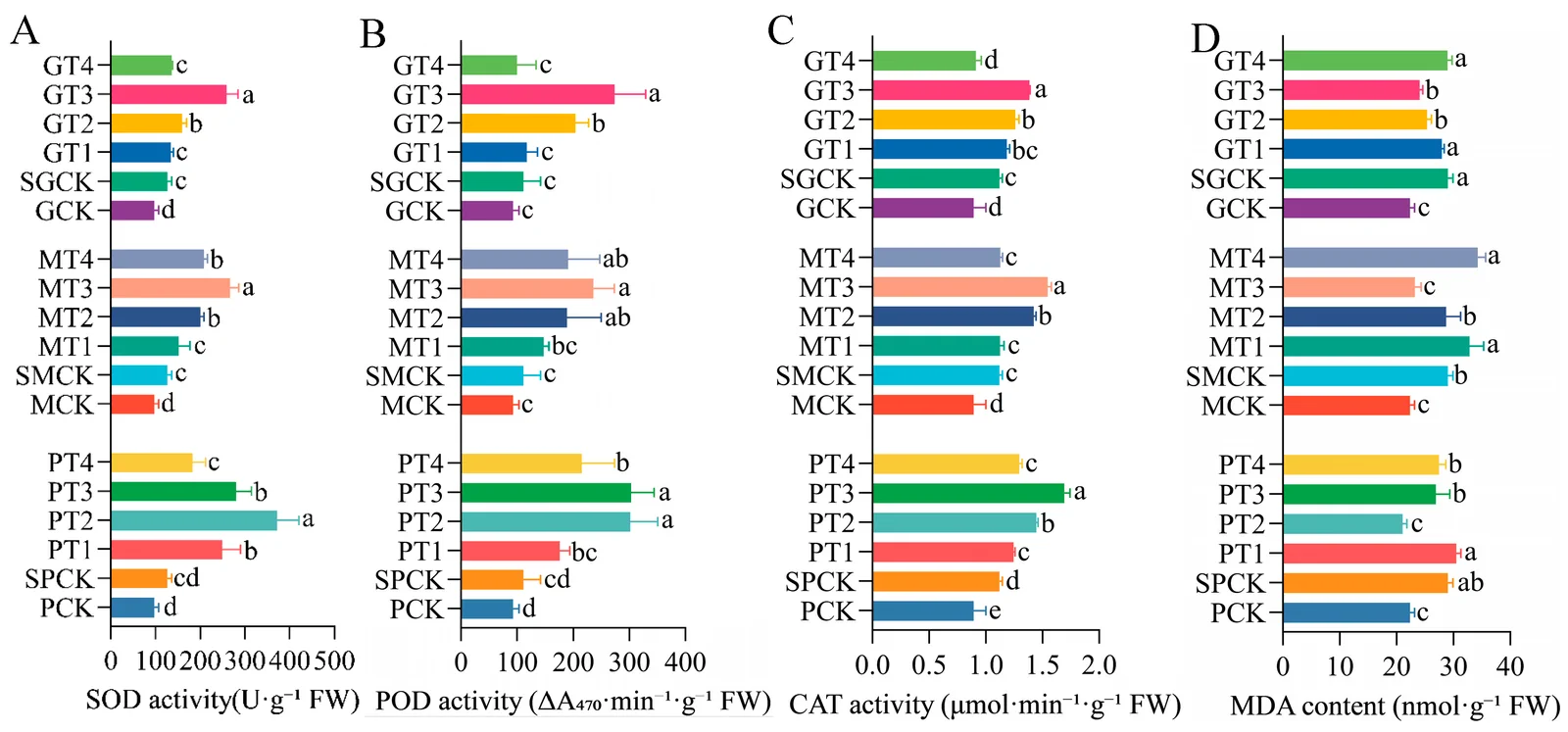
Effects of foliar PMO, MeJA, and GABA treatments on antioxidant enzyme activities and MDA content of sugar beet seedlings under NaCl stress. (**A**–**D**) Superoxide dismutase (SOD) activity, peroxidase (POD) activity, catalase (CAT) activity, and malondialdehyde (MDA) content, respectively. The treatments were grouped according to the three foliar treatments: PAA@Mn_3_O_4_ nanoparticles (PMO), methyl jasmonate (MeJA), and γ-aminobutyric acid (GABA). PCK, MCK, and GCK denote the same shared non-NaCl-stressed control (CK), whereas SPCK, SMCK, and SGCK denote the same shared NaCl-stressed control (SCK); these labels are repeated only to facilitate comparison within each treatment series and do not represent independent control groups. PT1–PT4, MT1–MT4, and GT1–GT4 represent four concentration treatments of PMO, MeJA, and GABA, respectively. Data are presented as mean ± SD from three independent experimental replicates (n = 3). Different lowercase letters indicate significant differences among treatments within the corresponding treatment series according to Duncan’s multiple range test (*p* < 0.05).

To evaluate the effects of foliar PMO, MeJA, and GABA treatments on antioxidant-related physiological responses under NaCl stress, the activities of superoxide dismutase (SOD), peroxidase (POD), catalase (CAT), and malondialdehyde (MDA) content were determined in sugar beet leaves. Compared with the shared non-NaCl-stressed control, NaCl treatment altered antioxidant enzyme activities and increased MDA accumulation. Foliar application of PMO, MeJA, and GABA further modified these responses, with treatment effects varying among substances and concentrations (Figure 7).

SOD is an antioxidant-related enzyme involved in superoxide metabolism. As shown in Figure 7A, leaf SOD activity significantly increased under NaCl stress compared with the non-NaCl-stressed control. Foliar application of PMO, MeJA, and GABA further increased SOD activity under NaCl stress, with a concentration-dependent pattern characterized by an initial increase followed by a decline. The highest SOD activity was observed under PT2, MT3, and GT3 treatments, increasing by 195.00%, 111.15%, and 105.58%, respectively, compared with the corresponding NaCl-stressed controls.

POD activity showed a similar response pattern (Figure 7B). NaCl stress alone did not significantly alter POD activity compared with the non-NaCl-stressed control, whereas foliar PMO, MeJA, and GABA treatments significantly increased POD activity. The highest POD activities within the respective treatment series were observed under PT3, MT3, and GT3 treatments, with increases of 171.56%, 111.03%, and 145.04%, respectively, relative to the corresponding NaCl-stressed controls.

CAT activity, another enzyme involved in hydrogen peroxide metabolism, was significantly increased by NaCl stress compared with the non-NaCl-stressed control (Figure 7C). Foliar application of PMO, MeJA, and GABA further enhanced CAT activity, with the highest values detected under PT3, MT3, and GT3 treatments. Compared with the corresponding NaCl-stressed controls, CAT activity increased by 51.49%, 37.96%, and 23.76%, respectively.

In contrast, MDA content exhibited an opposite response pattern (Figure 7D). NaCl treatment significantly increased MDA accumulation by 29.65% compared with the non-NaCl-stressed control, consistent with increased lipid peroxidation under the imposed stress condition. Foliar application of PMO, MeJA, and GABA significantly reduced MDA content, with the largest reductions observed under PT2, MT3, and GT3 treatments. Compared with the corresponding NaCl-stressed controls, MDA content decreased by 27.41%, 19.86%, and 16.91%, respectively.

Overall, PMO, MeJA, and GABA treatments were associated with enhanced antioxidant enzyme activities and reduced MDA accumulation under NaCl stress, suggesting improved antioxidant-related physiological responses in sugar beet seedlings.

### 2.4. Effects of Three Exogenous Substances on Osmolyte Accumulation in Sugar Beet Seedlings Under NaCl Stress

**Figure 8:**
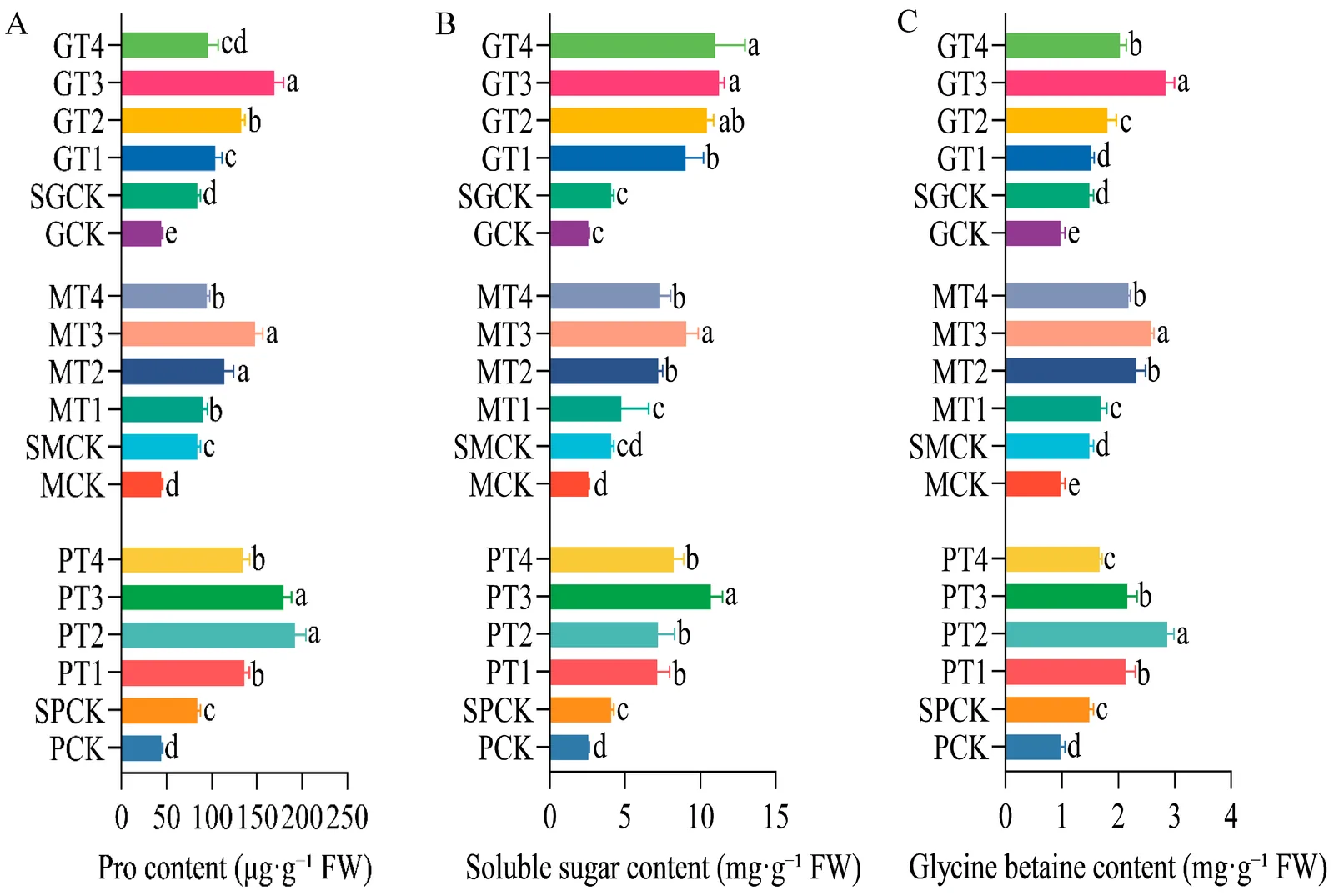
Effects of foliar PMO, MeJA, and GABA treatments on osmolyte contents in sugar beet seedlings under NaCl stress. (**A**–**C**) Proline (Pro) content, soluble sugar content, and glycine betaine content, respectively. The treatments were grouped according to the three foliar treatments: PAA@Mn_3_O_4_ nanoparticles (PMO), methyl jasmonate (MeJA), and γ-aminobutyric acid (GABA). PCK, MCK, and GCK denote the same shared non-NaCl-stressed control (CK), whereas SPCK, SMCK, and SGCK denote the same shared NaCl-stressed control (SCK); these labels are repeated only to facilitate comparison within each treatment series and do not represent independent control groups. PT1–PT4, MT1–MT4, and GT1–GT4 represent four concentration treatments of PMO, MeJA, and GABA, respectively. Data are presented as mean ± SD from three independent experimental replicates (n = 3). Different lowercase letters indicate significant differences among treatments within the corresponding treatment series according to Duncan’s multiple range test (*p* < 0.05).

To evaluate the effects of foliar PMO, MeJA, and GABA treatments on osmolyte-related responses of sugar beet seedlings under NaCl stress, the contents of three major osmolytes, including free proline, soluble sugar, and glycine betaine, were determined in leaves. NaCl stress significantly altered the accumulation patterns of these osmolytes, while foliar application of PMO, MeJA, and GABA further modified osmolyte levels, with responses varying among substances and concentrations (Figure 8).

Proline is an important osmolyte involved in plant responses to environmental stress. As shown in Figure 8A, free proline content in sugar beet leaves significantly increased under NaCl stress compared with the non-NaCl-stressed control. Foliar application of PMO, MeJA, and GABA further increased proline accumulation, although the response varied among concentrations and showed an initial increase followed by a decline. The highest proline contents were observed under PT2, MT3, and GT3 treatments, with increases of 129.41%, 76.95%, and 102.31%, respectively, compared with the corresponding NaCl-stressed controls.

Soluble sugar content showed a similar response pattern (Figure 8B). NaCl stress significantly increased soluble sugar accumulation in leaves, and foliar PMO, MeJA, and GABA treatments further promoted soluble sugar accumulation. The highest soluble sugar contents within the respective treatment series were observed under PT3, MT3, and GT3 treatments, which increased soluble sugar content by 162.58%, 122.21%, and 176.17%, respectively, compared with the corresponding NaCl-stressed controls.

Glycine betaine is another important compatible solute involved in plant stress responses. As shown in Figure 8C, NaCl stress significantly increased betaine content in sugar beet leaves. Foliar application of PMO, MeJA, and GABA further increased betaine accumulation, with the highest betaine contents observed under PT2, MT3, and GT3 treatments. Compared with the corresponding NaCl-stressed controls, betaine content increased by 92.69%, 73.31%, and 90.47%, respectively.

Overall, foliar PMO, MeJA, and GABA treatments were associated with altered accumulation of major osmolytes under NaCl stress, with response patterns differing among the three treatments.

### 2.5. Effects of Three Exogenous Substances on Selected Growth-Related Hormone Levels in Sugar Beet Seedlings

**Figure 9:**
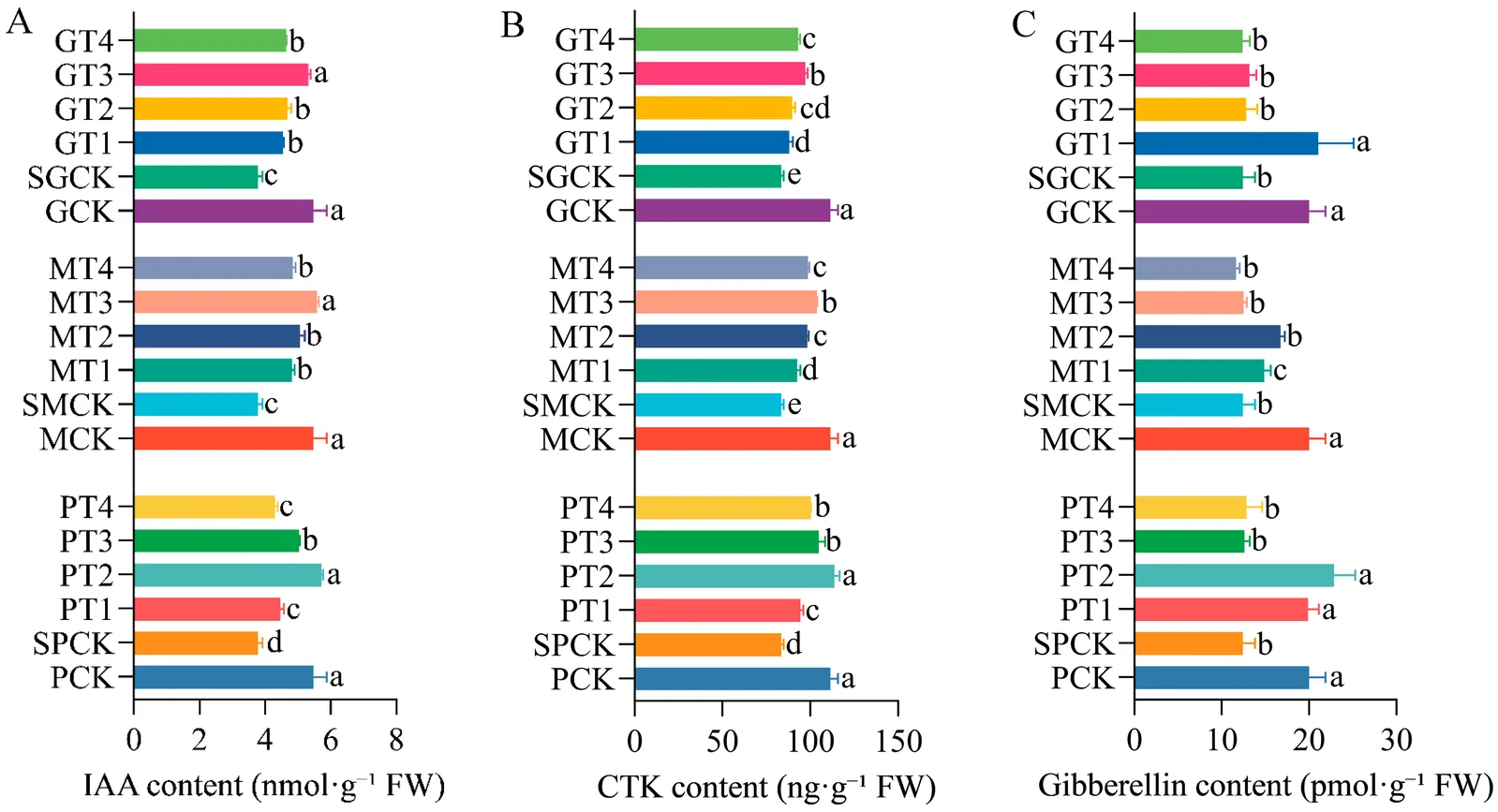
Effects of foliar PMO, MeJA, and GABA treatments on selected growth-related hormone levels in sugar beet seedlings under NaCl stress. (**A**–**C**) Indole-3-acetic acid (IAA) content, zeatin riboside (ZR; represented as CTK in the figures), and gibberellic acid (GA_3_; represented as GA in the figures), respectively. The treatments were grouped according to the three foliar treatments: PAA@Mn_3_O_4_ nanoparticles (PMO), methyl jasmonate (MeJA), and γ-aminobutyric acid (GABA). PCK, MCK, and GCK denote the same shared non-NaCl-stressed control (CK), whereas SPCK, SMCK, and SGCK denote the same shared NaCl-stressed control (SCK); these labels are repeated only to facilitate comparison within each treatment series and do not represent independent control groups. PT1–PT4, MT1–MT4, and GT1–GT4 represent four concentration treatments of PMO, MeJA, and GABA, respectively. Data are presented as mean ± SD from three independent experimental replicates (n = 3). Different lowercase letters indicate significant differences among treatments within the corresponding treatment series according to Duncan’s multiple range test (*p* < 0.05).

To evaluate the effects of foliar PMO, MeJA, and GABA treatments on growth-related hormone responses of sugar beet seedlings under NaCl stress, the levels of three endogenous hormones, including indole-3-acetic acid (IAA), zeatin riboside (ZR), and gibberellic acid (GA_3_), were determined in leaves. NaCl stress significantly altered the accumulation patterns of these hormones, while foliar application of PMO, MeJA, and GABA further modified hormone levels with different responses among treatments and concentrations (Figure 9).

IAA is an important growth-related hormone involved in the regulation of plant growth and development [22]. As shown in Figure 9A, NaCl stress significantly decreased IAA content in sugar beet leaves compared with the non-NaCl-stressed control. Foliar application of PMO, MeJA, and GABA increased IAA content under NaCl stress, although the response varied among concentrations and showed an initial increase followed by a decline. The highest IAA levels were observed under PT2, MT3, and GT3 treatments, increasing by 51.37%, 47.77%, and 40.68%, respectively, compared with the corresponding NaCl-stressed controls.

ZR, a cytokinin-related compound, is involved in regulating plant growth and developmental processes [23]. As shown in Figure 9B, NaCl stress significantly reduced ZR content in leaves. Foliar application of PMO, MeJA, and GABA increased ZR accumulation under NaCl stress, with the highest ZR contents observed under PT2, MT3, and GT3 treatments. Compared with the corresponding NaCl-stressed controls, ZR content increased by 36.13%, 24.34%, and 16.44%, respectively.

GA_3_ is an important growth-related hormone associated with plant development [24]. As shown in Figure 9C, NaCl stress significantly decreased GA_3_ content in sugar beet leaves. Foliar application of PMO, MeJA, and GABA increased GA_3_ levels to varying degrees, with concentration-dependent variations among treatments. The highest GA_3_ contents were observed under PT2, MT2, and GT1 treatments, which increased by 83.83%, 34.57%, and 69.33%, respectively, compared with the corresponding NaCl-stressed controls.

Overall, foliar PMO, MeJA, and GABA treatments were associated with changes in selected growth-related hormone levels under NaCl stress, with response patterns differing among the three foliar treatments.

### 2.6. Effects of Three Exogenous Substances on Storage-Root Yield, Estimated Storage-Root Sugar Concentration, and Estimated Sugar Yield of Sugar Beet Under Non-Saline–Alkali and Saline–Alkali Field Conditions

**Figure 10:**
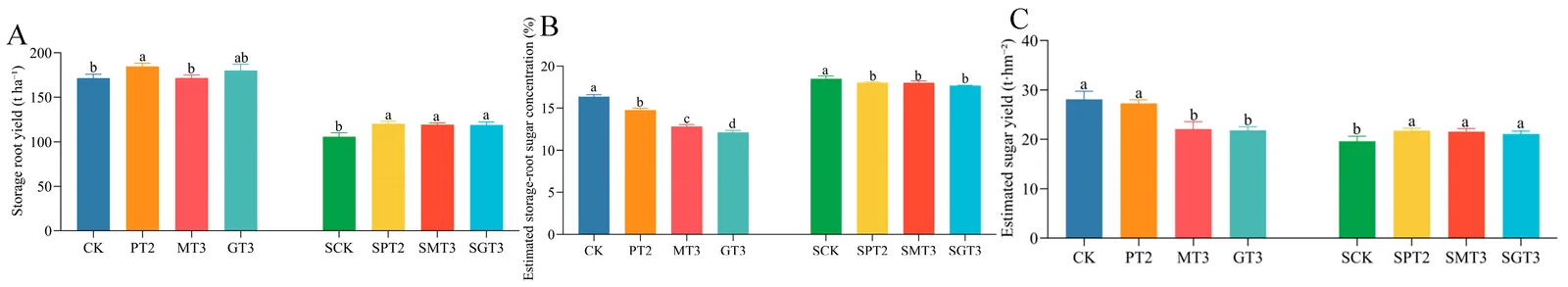
Effects of three exogenous substances on storage-root yield, soluble solids content, and estimated sugar yield of sugar beet under non-saline–alkali and saline–alkali field conditions. (**A**) Storage-root yield; (**B**) estimated storage-root sugar concentration; and (**C**) estimated sugar yield. The field experiment evaluated the selected concentrations from the preceding pot experiment under two contrasting field conditions: non-saline–alkali soil and saline–alkali soil. CK, PT2, MT3, and GT3 represent the ultrapure water-sprayed control, 100 mg L^−1^ PAA@Mn_3_O_4_ nanoparticle (PMO) treatment, 100 mg L^−1^ methyl jasmonate (MeJA) treatment, and 1000 mg L^−1^ γ-aminobutyric acid (GABA) treatment under non-saline–alkali soil conditions, respectively. SCK, SPT2, SMT3, and SGT3 represent the corresponding treatments under saline–alkali soil conditions, respectively. Data are presented as mean ± SD from three independent plots (n = 3). Different lowercase letters indicate significant differences among treatments within the same field condition according to Duncan’s multiple range test (*p* < 0.05).

To evaluate the field performance of the concentrations selected from the preceding pot experiment, a one-season field experiment was conducted under non-saline–alkali and saline–alkali soil conditions (Figure 10). The effects of 100 mg L^−1^ PAA@Mn_3_O_4_ nanoparticles (PT2), 100 mg L^−1^ methyl jasmonate (MT3), and 1000 mg L^−1^ γ-aminobutyric acid (GT3) on storage-root yield, estimated storage-root sugar concentration, and estimated sugar yield were evaluated separately within each field condition.

Under non-saline–alkali soil conditions, only PT2 significantly increased storage-root yield by 7.66% compared with CK, whereas MT3 and GT3 did not differ significantly from the control (Figure 10A). The estimated storage-root sugar concentration was significantly lower in all three treatments than in CK (Figure 10B). Consequently, the estimated sugar yield of PT2 was not significantly different from that of CK, whereas MT3 and GT3 significantly reduced estimated sugar yield (Figure 10C).

Under saline–alkali soil conditions, SPT2, SMT3, and SGT3 significantly increased storage-root yield by 13.59%, 12.69%, and 12.37%, respectively, compared with SCK (Figure 10A). Although the estimated storage-root sugar concentration was significantly lower in all three treatments than in SCK (Figure 10B), the corresponding estimated sugar yields remained significantly higher because of the greater storage-root yields. Compared with SCK, estimated sugar yield increased by 10.77%, 9.79%, and 7.41% in SPT2, SMT3, and SGT3, respectively (Figure 10C).

### 2.7. Effects of Three Exogenous Substances on Dry Matter Accumulation and Biomass Allocation of Sugar Beet Under Field Conditions

**Figure 11:**
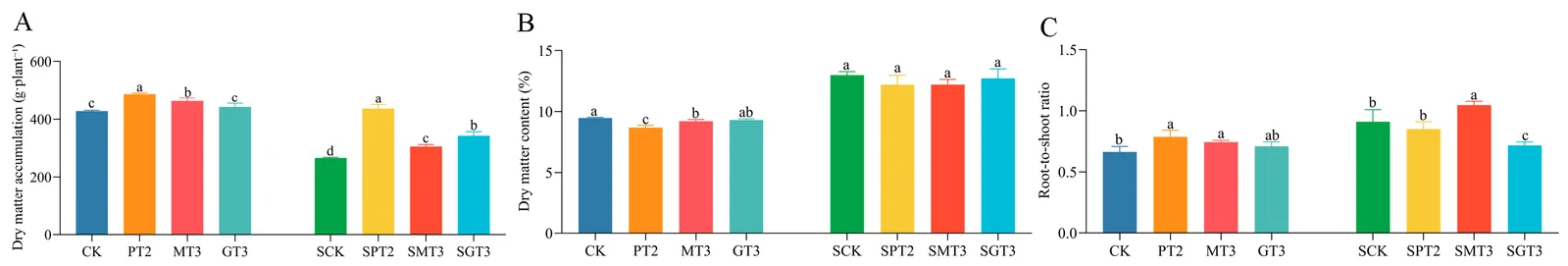
Effects of foliar PMO, MeJA, and GABA treatments on dry matter accumulation, dry matter content, and root-to-shoot ratio of sugar beet under non-saline–alkali and saline–alkali field conditions. (**A**) Dry matter accumulation per plant; (**B**) dry matter content; and (**C**) root-to-shoot ratio. CK, PT2, MT3, and GT3 represent the ultrapure water-sprayed control, 100 mg L^−1^ PAA@Mn_3_O_4_ nanoparticle (PMO) treatment, 100 mg L^−1^ methyl jasmonate (MeJA) treatment, and 1000 mg L^−1^ γ-aminobutyric acid (GABA) treatment under non-saline–alkali soil conditions, respectively. SCK, SPT2, SMT3, and SGT3 represent the corresponding treatments under saline–alkali soil conditions, respectively. Data are presented as mean ± SD from three independent plots (n = 3). Different lowercase letters indicate significant differences among treatments within the same field condition according to Duncan’s multiple range test (*p* < 0.05).

To evaluate the effects of foliar PMO, MeJA, and GABA treatments on dry matter accumulation and biomass allocation of sugar beet under field conditions, dry matter accumulation per plant, dry matter content, and root-to-shoot ratio were determined under two contrasting field conditions: non-saline–alkali soil and saline–alkali soil (Figure 11). Different response patterns were observed at the two field sites.

Under saline–alkali soil conditions, all three foliar treatments significantly increased dry matter accumulation per plant compared with SCK (Figure 11A). PT2, MT3, and GT3 treatments increased dry matter accumulation by 64.62%, 15.38%, and 29.49%, respectively. No significant differences in dry matter content were observed between the treatments and SCK (Figure 11B). For root-to-shoot ratio (Figure 11C), only MT3 significantly increased this parameter compared with SCK, with an increase of 14.76%, whereas PT2 and GT3 showed no significant differences.

### 2.8. Effects of Three Exogenous Substances on Antioxidant Enzyme Activities and Malondialdehyde Content in Leaves and Roots of Sugar Beet

To evaluate the effects of foliar PMO, MeJA, and GABA treatments on antioxidant-related physiological traits under field conditions, the activities of superoxide dismutase (SOD), peroxidase (POD), catalase (CAT), and MDA content were determined in leaves and roots of sugar beet grown under two contrasting field conditions: non-saline–alkali soil and saline–alkali soil.

**Figure 12:**
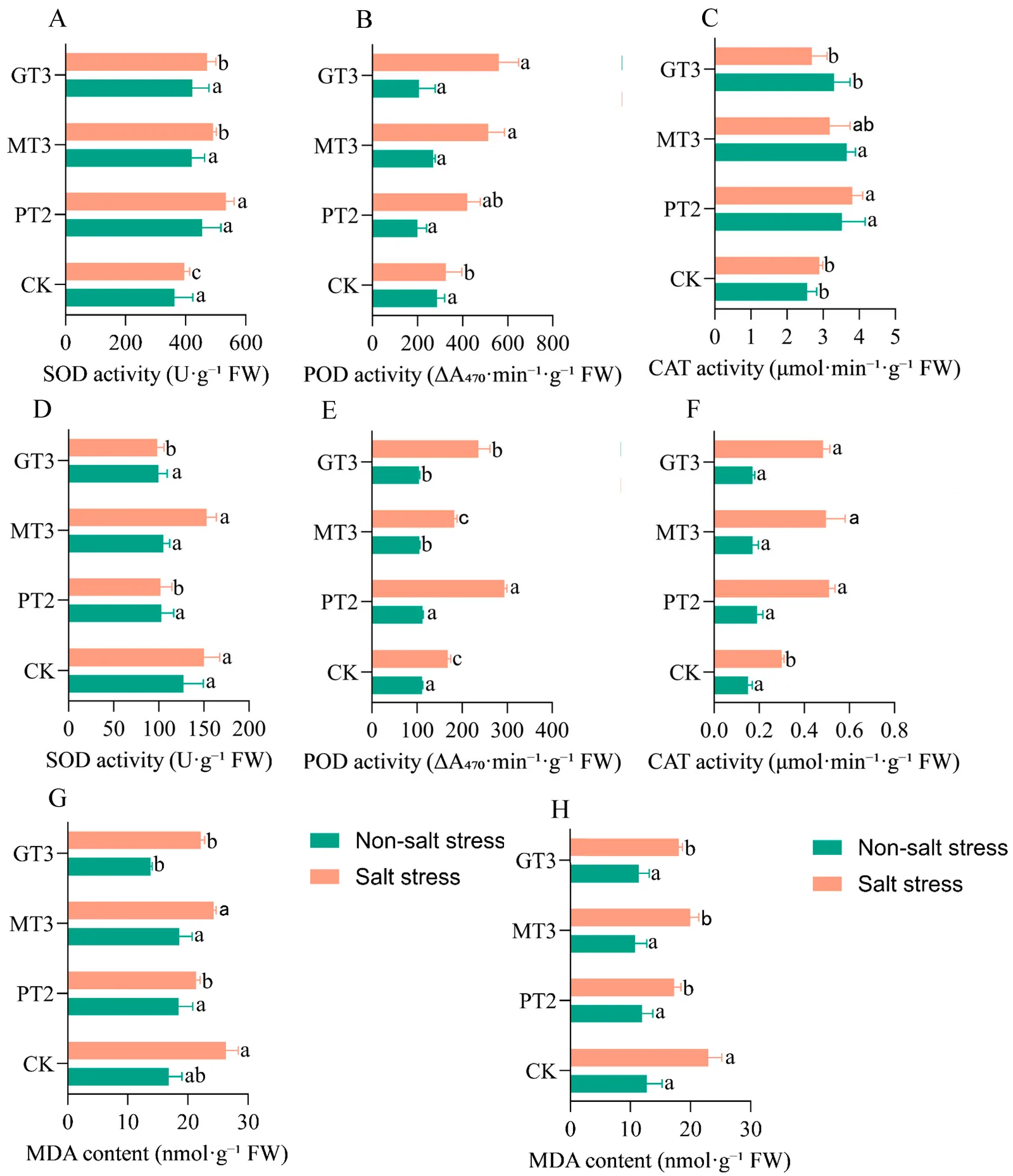
Effects of foliar PMO, MeJA, and GABA treatments on antioxidant enzyme activities and MDA content in leaves and roots of sugar beet under non-saline–alkali and saline–alkali field conditions. CK represents the ultrapure water-sprayed control treatment. PT2, MT3, and GT3 represent the 100 mg L^−1^ PAA@Mn_3_O_4_ nanoparticle (PMO) treatment, 100 mg L^−1^ methyl jasmonate (MeJA) treatment, and 1000 mg L^−1^ γ-aminobutyric acid (GABA) treatment, respectively. Green bars represent non-saline–alkali soil conditions, and orange bars represent saline–alkali soil conditions. Panels (**A**–**C**,**G**) show leaf measurements, whereas panels (**D**–**F**,**H**) show root measurements. Panels (**A**,**D**) represent superoxide dismutase (SOD) activity; panels (**B**,**E**) represent peroxidase (POD) activity; panels (**C**,**F**) represent catalase (CAT) activity; and panels (**G**,**H**) represent malondialdehyde (MDA) content. Data are presented as mean ± SD from three independent plots (n = 3). Different lowercase letters indicate significant differences among treatments within the same field condition and organ according to Duncan’s multiple range test (*p* < 0.05).

Different response patterns of leaf antioxidant-related traits were observed at the two field sites (Figure 12A–C,G). Under non-saline–alkali soil conditions, no significant differences were observed in leaf SOD and POD activities or MDA content between the foliar treatments and CK. Only PT2 significantly increased CAT activity by 23.15% compared with CK.

Under saline–alkali soil conditions, foliar PMO, MeJA, and GABA treatments altered leaf antioxidant-related traits to varying degrees. Compared with SCK, PT2, MT3, and GT3 increased SOD activity by 35.05%, 24.28%, and 19.36%, respectively. MT3 and GT3 treatments significantly increased POD activity by 57.95% and 72.28%, respectively, whereas PT2 and MT3 treatments increased CAT activity by 31.22% and 9.71%, respectively. In addition, leaf MDA content was significantly reduced by 23.12% under PT2 and by 13.68% under GT3 compared with SCK.

Root antioxidant-related traits showed different response patterns from those observed in leaves (Figure 12D–F,H). Under non-saline–alkali soil conditions, no significant differences were observed in root SOD and CAT activities or MDA content between the treatments and the control. Only POD activity under MT3 and GT3 treatments showed significant reductions of 5.85% and 6.68%, respectively, compared with the control.

Under saline–alkali soil conditions, PMO, MeJA, and GABA treatments were associated with different changes in storage-root antioxidant-related traits. Compared with the control, PT2 and GT3 treatments significantly decreased SOD activity by 47.09% and 55.64%, respectively, whereas MT3 showed no significant difference. POD activity was significantly increased by 74.39% and 40.23% under PT2 and GT3 treatments, respectively. CAT activity was significantly increased under PT2, MT3, and GT3 treatments, with increases of 70.17%, 65.06%, and 59.98%, respectively. Meanwhile, root MDA content was significantly reduced by 33.18%, 15.11%, and 15.63% under PT2, MT3, and GT3 treatments, respectively, compared with the control.

### 2.9. Effects of Three Exogenous Substances on Photosynthetic Characteristics of Sugar Beet Leaves

**Figure 13:**
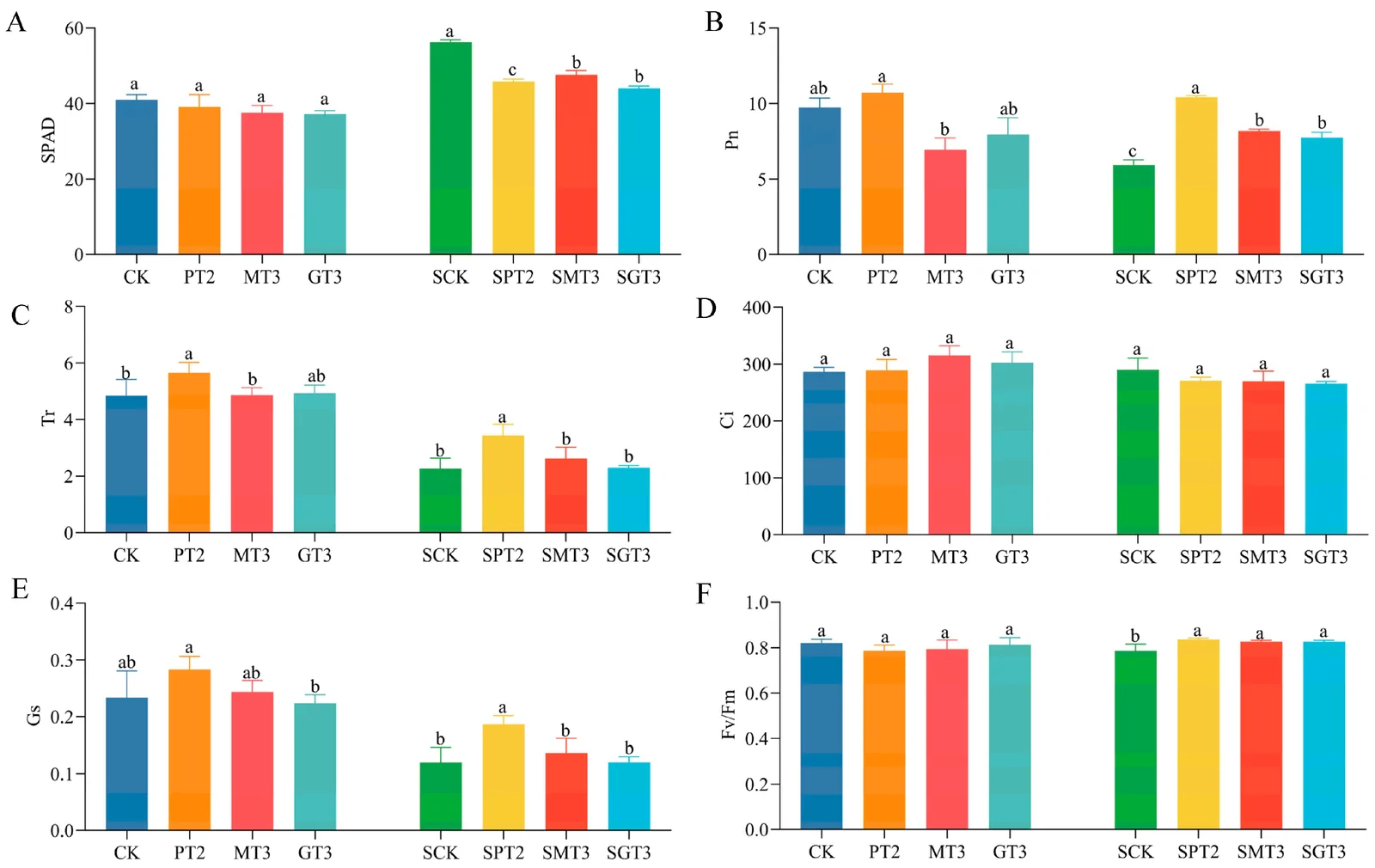
Effects of foliar PMO, MeJA, and GABA treatments on photosynthetic characteristics of sugar beet leaves under non-saline–alkali and saline–alkali field conditions. (**A**) Leaf SPAD value; (**B**) net photosynthetic rate (Pn); (**C**) transpiration rate (Tr); (**D**) intercellular CO_2_ concentration (Ci); (**E**) stomatal conductance (Gs); and (**F**) maximum photochemical efficiency of PSII (Fv/Fm). CK, PT2, MT3, and GT3 represent the ultrapure water-sprayed control, 100 mg L^−1^ PAA@Mn_3_O_4_ nanoparticle (PMO) treatment, 100 mg L^−1^ methyl jasmonate (MeJA) treatment, and 1000 mg L^−1^ γ-aminobutyric acid (GABA) treatment under non-saline–alkali soil conditions, respectively. SCK, SPT2, SMT3, and SGT3 represent the corresponding treatments under saline–alkali soil conditions, respectively. Data are presented as mean ± SD from three independent plots (n = 3). Different lowercase letters indicate significant differences among treatments within the same field condition according to Duncan’s multiple range test (*p* < 0.05).

To evaluate the effects of foliar PMO, MeJA, and GABA treatments on photosynthetic characteristics of sugar beet leaves, leaf SPAD value, gas-exchange parameters, and chlorophyll fluorescence parameters were determined under two contrasting field conditions: non-saline–alkali soil and saline–alkali soil (Figure 13).

Under non-saline–alkali conditions, no significant differences were observed in SPAD value, net photosynthetic rate (Pn), intercellular CO_2_ concentration (Ci), and stomatal conductance (Gs) between PT2, MT3, and GT3 treatments and the control, except that PT2 significantly increased transpiration rate (Tr) by 51.99% compared with CK (Figure 12A–E). No significant differences in Fv/Fm were observed among treatments under non-saline–alkali conditions (Figure 13F).

Under saline–alkali conditions, the responses of photosynthetic traits varied among treatments. Compared with SCK, SPT2, SMT3, and SGT3 treatments significantly reduced leaf SPAD values by 22.93%, 18.11%, and 27.95%, respectively (Figure 13A). In contrast, net photosynthetic rate (Pn) was significantly increased under SPT2, SMT3, and SGT3 treatments, with increases of 75.60%, 37.64%, and 30.64%, respectively, and the greatest increase was observed under SPT2 treatment (Figure 13B).

For Tr (Figure 13C), only SPT2 treatment significantly increased this parameter by 16.47% compared with SCK, whereas SMT3 and SGT3 showed no significant differences. Intercellular CO_2_ concentration (Ci) remained unchanged among treatments under both field conditions (Figure 13D). For stomatal conductance (Gs), no significant differences were observed among treatments under non-saline–alkali conditions, whereas SPT2 significantly increased Gs by 21.74% compared with SCK under saline–alkali conditions (Figure 13E).

For the chlorophyll fluorescence parameter Fv/Fm (Figure 13F), no significant differences were detected among treatments under non-saline–alkali conditions. Under saline–alkali conditions, SPT2, SMT3, and SGT3 significantly increased Fv/Fm by 6.18%, 4.50%, and 4.44%, respectively, compared with SCK.

## 3. Discussion

Soil salinization is a major limiting factor affecting crop productivity in salt-affected agricultural regions, and the application of exogenous substances has become an important approach for improving crop performance under adverse environmental conditions [25]. In this study, pot-based concentration screening and subsequent field evaluation were combined to comparatively evaluate the effects of three foliar-applied substances with different physiological functions, namely PAA@Mn_3_O_4_ nanoparticles (PMO), methyl jasmonate (MeJA), and γ-aminobutyric acid (GABA), on sugar beet responses under salt-affected conditions. The pot experiment was first conducted under controlled NaCl stress to identify concentrations for subsequent field evaluation, followed by a field experiment under contrasting non-saline–alkali and saline–alkali soil conditions to evaluate their agronomic responses under field conditions.

The results showed that PMO, MeJA, and GABA treatments were associated with different physiological and growth-related responses under the imposed NaCl treatment and field saline–alkali conditions. In the pot experiment, the three substances affected multiple physiological traits, including growth characteristics, photosynthetic performance, antioxidant-related traits, osmolyte accumulation, and selected growth-related hormone levels. Based on the overall response patterns across the measured growth and physiological traits, 100 mg L^−1^ PMO, 100 mg L^−1^ MeJA, and 1000 mg L^−1^ GABA were selected for subsequent field evaluation. Under saline–alkali field conditions, these selected treatments increased storage-root yield and estimated sugar yield, supporting their further agronomic evaluation under salt-affected production conditions. However, the observed changes in physiological traits should not be interpreted as direct evidence of specific molecular or nanoparticle-specific mechanisms.

### 3.1. Effects of Three Exogenous Substances on Plant Growth and Biomass Accumulation

Salt stress negatively affects plant growth by disturbing water balance, nutrient uptake, photosynthetic activity, and biomass allocation [26]. In the present study, controlled NaCl stress significantly inhibited sugar beet seedling growth, as indicated by reductions in leaf number, root architectural traits, and dry matter accumulation. These results are consistent with previous reports showing that early growth stages of sugar beet are sensitive to saline conditions.

Foliar application of PMO, MeJA, and GABA was associated with improvements in several growth-related traits of sugar beet seedlings subjected to the imposed NaCl treatment. In the pot experiment, the selected concentrations of the three substances increased dry matter accumulation and improved several growth-related traits compared with the salt-stressed control. These results indicate that the three foliar treatments were associated with improved growth performance under the imposed NaCl treatment.

PMO treatment showed pronounced responses in several biomass- and yield-related traits. In the pot experiment, PT2 (100 mg L^−1^ PMO), which was subsequently selected for field evaluation, increased dry matter accumulation by 64.80% compared with the corresponding NaCl-stressed control. Under saline–alkali field conditions, PMO treatment increased storage-root yield by 13.59% relative to SCK. Previous studies have reported that Mn-based nanomaterials can influence plant growth and stress-related physiological processes under adverse environments [27,28]. However, the present study focused on physiological responses following PMO application and did not directly evaluate nanozyme activity, ROS-scavenging capacity, or nanoparticle transport characteristics in sugar beet. Therefore, the observed growth responses following PMO treatment should be interpreted as integrated physiological responses rather than direct evidence of a specific nanomaterial-mediated mechanism.

MeJA and GABA also improved growth-related traits under salt stress and saline–alkali field conditions. Previous studies have demonstrated that MeJA participates in plant stress signaling and regulates physiological processes under adverse environments, whereas GABA is involved in metabolic regulation during stress responses [29,30,31,32]. The responses observed following MeJA and GABA application support their further agronomic evaluation under salt-affected production conditions.

Importantly, the pot and field experiments provided different types of evidence. The pot experiment mainly supported concentration screening and physiological evaluation under controlled NaCl stress, whereas the field experiment provided preliminary evidence of agronomic performance under the two field conditions evaluated. The responses observed in the two experiments provide complementary rather than equivalent evidence, because physiological responses under controlled NaCl stress cannot be assumed to directly predict field performance.

### 3.2. Effects of Three Exogenous Substances on Photosynthetic Performance

Photosynthesis is highly sensitive to salt stress, and maintaining photosynthetic activity is essential for sustaining plant growth under saline conditions [32]. In this study, PMO, MeJA, and GABA treatments influenced photosynthetic-related traits in sugar beet under both controlled NaCl stress and saline–alkali field conditions.

In the pot experiment, the three treatments increased Fv/Fm, ΦPSII, and qL to varying degrees and modified NPQ responses under the imposed NaCl treatment. These changes indicate that the treatments were associated with altered PSII photochemical performance under the imposed NaCl treatment.

Under saline–alkali field conditions, PMO treatment significantly increased net photosynthetic rate (Pn) by 75.60% compared with SCK. MeJA and GABA treatments also significantly increased Pn, although the magnitude of the observed responses differed among treatments. Previous studies have reported changes in photosynthetic performance following the application of Mn-based nanomaterials under environmental stress conditions [33]. In the present study, PMO treatment was likewise associated with changes in several measured photosynthetic traits.

Under saline–alkali field conditions, Pn increased following all three foliar treatments, whereas Gs and Fv/Fm showed treatment-dependent responses and Ci did not differ significantly among treatments. These parameters were therefore interpreted jointly as measured photosynthetic responses, without attributing the observed changes in Pn to a specific stomatal or non-stomatal mechanism.

MeJA has been reported to regulate stress responses and photosynthetic performance in different crops [34,35,36,37]. In this study, MeJA treatment was associated with changes in several photosynthetic parameters under saline–alkali conditions, broadly consistent with previous reports of MeJA-related physiological responses under adverse conditions. Similarly, GABA has been reported to participate in plant adaptation to abiotic stress through metabolic regulation and cellular responses [34,38].

### 3.3. Effects of Three Exogenous Substances on Antioxidant-Related Responses

Salt stress induces excessive reactive oxygen species (ROS) accumulation, resulting in oxidative imbalance and membrane lipid peroxidation, which is an important factor limiting plant growth under saline conditions [39]. Plants respond to oxidative stress through coordinated regulation of antioxidant enzymes, including superoxide dismutase (SOD), peroxidase (POD), and catalase (CAT). In this study, PMO, MeJA, and GABA treatments modified antioxidant-related physiological responses in sugar beet under controlled NaCl stress and saline–alkali field conditions [40].

In the pot experiment, salt stress significantly altered antioxidant enzyme activities and increased MDA accumulation in sugar beet leaves. Foliar application of PMO, MeJA, and GABA further modified these responses, with increased activities of SOD, POD, and CAT and reduced MDA content observed under selected concentrations. These results indicate that the three treatments were associated with changes in antioxidant-related traits under the imposed NaCl treatment.

PMO treatment was associated with a pronounced reduction in MDA accumulation at the selected concentration. Under PT2 (100 mg L^−1^ PMO), which was selected for subsequent field evaluation, leaf MDA content decreased by 27.41% compared with the corresponding NaCl-stressed control. Previous studies have reported that Mn-based nanomaterials can influence antioxidant-related physiological responses in plants under environmental stress [12,41]. However, the present study did not directly determine ROS accumulation, nanozyme catalytic activity, or PMO-mediated ROS-scavenging capacity. Therefore, the reduction in MDA content and changes in antioxidant enzyme activities observed after PMO application should be interpreted as physiological responses associated with PMO treatment rather than direct evidence of ROS elimination by PMO.

MeJA and GABA also influenced antioxidant-related traits under salt stress. Previous studies have shown that MeJA participates in plant stress signaling and antioxidant metabolism regulation, whereas GABA contributes to stress-associated metabolic adjustment [42,43,44,45]. Consistent with previous findings, MeJA and GABA treatments were associated with changes in antioxidant enzyme activities and MDA accumulation in sugar beet seedlings under the imposed NaCl treatment.

The field experiment further showed different antioxidant-related response patterns in leaves and storage roots. Under saline–alkali conditions, leaf antioxidant-related traits generally showed increased enzyme activities and reduced MDA accumulation after foliar treatments. However, storage-root responses showed distinct patterns, with some treatments decreasing SOD activity while increasing POD and CAT activities and reducing MDA content. These results indicate that antioxidant-related responses were tissue-dependent and cannot be simply described as universal enhancement of all antioxidant enzymes.

Leaves and storage roots showed different antioxidant-related response patterns under field conditions. These tissue-specific differences should be interpreted descriptively because ROS levels, antioxidant-related gene expression, and other mechanistic indicators were not directly measured in the present study. Further studies involving ROS detection, antioxidant gene expression analysis, and biochemical characterization are required to clarify the detailed regulatory mechanisms.

### 3.4. Effects of Three Exogenous Substances on Osmolyte Accumulation

Osmotic imbalance is one of the major physiological challenges caused by salt stress. Plants commonly accumulate compatible solutes, including proline, soluble sugars, and glycine betaine, which participate in osmotic regulation and metabolic protection under adverse environments [46]. In this study, salt stress altered osmolyte accumulation patterns in sugar beet leaves, and foliar application of PMO, MeJA, and GABA further modified these responses.

In the pot experiment, NaCl stress significantly increased free proline, soluble sugar, and glycine betaine contents, reflecting altered osmolyte accumulation in sugar beet seedlings. Foliar application of PMO, MeJA, and GABA further promoted the accumulation of these osmolytes, although the response patterns differed among treatments and concentrations.

PMO treatment was associated with pronounced changes in several osmolyte-related traits. Under PT2 (100 mg L^−1^ PMO), which was selected for subsequent field evaluation, proline and glycine betaine contents increased by 129.41% and 92.69%, respectively, compared with the corresponding NaCl-stressed control. Previous studies have demonstrated that proline, soluble sugars, and glycine betaine contribute to plant adaptation to osmotic stress through regulation of cellular osmotic conditions and protection of metabolic processes [47,48].

However, increased osmolyte accumulation should not be interpreted simply as enhanced stress tolerance. The accumulation of osmolytes may represent both adaptive responses and metabolic adjustments triggered by stress exposure. Therefore, the increased levels of proline, soluble sugars, and glycine betaine observed after foliar treatments in this study reflect altered osmolyte metabolism associated with salt stress responses rather than direct evidence of improved osmotic adjustment capacity.

The differences observed among PMO, MeJA, and GABA treatments indicate that their effects on osmolyte accumulation were not identical. Further metabolomic and molecular studies are needed to clarify the processes associated with treatment-related changes in osmolyte biosynthesis and metabolism under saline conditions.

### 3.5. Effects of Three Exogenous Substances on Growth-Related Hormone Levels

Plant hormones play essential roles in regulating growth, development, and stress responses [49]. Salt stress often alters endogenous hormone metabolism, resulting in changes in growth-related hormone levels and affecting plant developmental processes [50]. In this study, the effects of PMO, MeJA, and GABA treatments on selected growth-related hormones, including indole-3-acetic acid (IAA), zeatin riboside (ZR), and gibberellic acid (GA_3_), were evaluated in sugar beet leaves under controlled NaCl stress.

NaCl stress significantly decreased IAA, ZR, and GA_3_ contents in sugar beet leaves, whereas foliar application of PMO, MeJA, and GABA increased these hormone levels to different degrees. The responses generally showed a concentration-dependent variation, with the highest values usually observed at intermediate concentrations rather than continuously increasing with application rates.

PMO treatment was associated with pronounced changes in selected growth-related hormone levels. Under PT2 (100 mg L^−1^ PMO), which was selected for subsequent field evaluation, IAA, ZR, and GA_3_ contents increased compared with the corresponding NaCl-stressed control. Previous studies have reported that Mn-based materials and other exogenous substances may influence plant growth-related physiological processes under stress conditions. However, the present study only measured changes in hormone levels and did not determine hormone biosynthesis pathways, transport processes, or signaling components.

MeJA and GABA also increased selected hormone levels under salt stress. Previous studies have shown that MeJA participates in the coordination of plant growth and stress-related responses, while GABA is involved in metabolic regulation under adverse conditions [26,27,28,29,30]. The observed changes in hormone levels after MeJA and GABA treatments were associated with corresponding changes in growth-related physiological traits under the imposed NaCl treatment.

Overall, the changes in IAA, ZR, and GA_3_ levels observed in this study indicate that PMO, MeJA, and GABA treatments were associated with altered growth-related hormone responses under salt stress. However, these changes should be interpreted as treatment-associated physiological responses rather than direct evidence of hormone-mediated regulation or changes in hormone homeostasis.

### 3.6. Relationship Between Controlled Salt-Stress Responses and Field Saline–Alkali Performance

The pot and field experiments in this study were designed to provide complementary rather than equivalent evidence regarding sugar beet responses under different salt-affected conditions. The pot experiment was conducted under controlled NaCl stress and was primarily used to identify concentrations of PMO, MeJA, and GABA for subsequent field evaluation and to characterize associated early physiological responses of sugar beet seedlings.

Under the imposed NaCl treatment, foliar application of the three substances affected multiple measured traits, including growth characteristics, photosynthetic performance, antioxidant-related responses, osmolyte accumulation, and selected growth-related hormone levels. These response patterns were considered when selecting concentrations for subsequent field evaluation.

Field conditions, however, are more complex than controlled pot conditions and involve variation in soil properties, environmental conditions, and crop growth duration. Accordingly, field performance reflects the integrated outcome of plant development and yield formation under site-specific conditions rather than a direct extension of the responses observed in the pot experiment.

The selected concentrations showed positive yield-related responses under the saline–alkali field condition. PMO, MeJA, and GABA treatments increased storage-root yield and estimated sugar yield relative to the corresponding control. However, the magnitude and pattern of responses differed between the pot and field experiments, indicating that physiological responses observed under controlled NaCl stress cannot be assumed to directly predict field performance.

Therefore, the pot experiment should be interpreted as a concentration-screening and physiological assessment approach, whereas the field experiment provides preliminary evidence of agronomic performance under the two field conditions evaluated in this study. Together, these two experimental approaches provide complementary information for further evaluation of foliar PMO, MeJA, and GABA treatments under salt-affected production conditions.

### 3.7. Limitations and Future Perspectives

Several limitations should be considered when interpreting the present results. First, nanoparticle uptake, translocation, and accumulation in leaves, roots, storage roots, and soil were not directly measured. Therefore, the present study cannot establish the fate of foliar-applied PMO within the plant–soil system or attribute the observed physiological responses to a specific nanoparticle transport pathway. In addition, tissue concentrations of Mn, Na^+^, K^+^, Cl^−^, and other mineral elements were not determined, limiting interpretation of possible changes in ion balance or mineral nutrition.

Second, nanoparticle-specific control treatments, including equivalent Mn^2+^, PAA-only, bulk Mn_3_O_4_, and particle-free supernatant controls, were not included. Accordingly, the observed responses cannot be attributed specifically to the nanoscale properties of PMO, its PAA coating, or Mn_3_O_4_ itself. Furthermore, the specific PMO batch used in this study was not independently characterized for particle size, aggregation behavior, surface charge, dissolved Mn release, or stability in the final foliar formulation.

Finally, the field experiment was conducted for one growing season at two sites, with each soil condition represented by a single site. Therefore, the field results should be considered preliminary and require further evaluation across additional years and locations. Environmental fate, nanoparticle persistence or transformation, soil accumulation, runoff, crop residue safety, occupational exposure, and the feasibility of repeated field applications were also not evaluated. Consequently, the present study does not provide sufficient evidence to draw conclusions regarding environmental safety or readiness for broad agricultural deployment. These aspects should be addressed in future studies before wider agronomic use is considered.

## 4. Materials and Methods

### 4.1. Experimental Materials

#### 4.1.1. Plant Material

The sugar beet (*Beta vulgaris* L.) cultivar ‘HI0479’ was used as the test material, which was provided by Syngenta Group Co., Ltd. (Basel, Switzerland).

#### 4.1.2. Reagents

The exogenous regulatory substances and key reagents used in this study are as follows: γ-aminobutyric acid (GABA; purity ≥ 99%; CAS No. 56-12-2) and methyl jasmonate (MeJA; purity ≥98%; CAS No. 39924-52-2), both purchased from Shanghai Macklin Biochemical Co., Ltd. (Shanghai, China); poly(acrylic acid)-coated Mn_3_O_4_ nanoparticles (PAA@Mn_3_O_4_), commonly abbreviated as PMO in the literature, provided by the research group of Prof. Honghong Wu at Huazhong Agricultural University; sodium chloride (NaCl, analytical grade), purchased from Tianjin Zhiyuan Chemical Reagent Co., Ltd. (Tianjin, China) and used for salt stress treatment.

The PMO material is a well-established nanosystem previously developed by Prof. Wu’s group for plant abiotic stress research, with detailed synthesis, purification and physicochemical characterization protocols fully reported in prior publications. Briefly, MnSO_4_·H_2_O was used as the manganese precursor and poly(acrylic acid) (PAA) as the surface coating agent. After hydrothermal synthesis, large particles and aggregates were removed via centrifugation, followed by further purification through dialysis and membrane filtration [12,41].

This PMO system has been systematically characterized in previous studies using high-resolution transmission electron microscopy (HRTEM), dynamic light scattering (DLS), zeta potential analysis, X-ray diffraction (XRD), and X-ray photoelectron spectroscopy (XPS). The reported HRTEM primary particle size is 5.6 ± 1.1 nm, with a hydrodynamic diameter of 9.2 ± 1.2 nm measured by DLS and a zeta potential of −38.3 ± 0.1 mV. XRD analysis confirms the pure Mn_3_O_4_ crystalline phase, and XPS demonstrates the coexistence of Mn^2+^, Mn^3+^, and Mn^4+^ at an approximate ratio of 0.4:1:1.9. In addition, in vitro activity assays verify that PMO possesses reactive oxygen species (ROS)-scavenging capacity, with scavenging efficiencies of 95.1%, 30.0%, and 6.7% for •OH, O_2_•^−^, and H_2_O_2_, respectively [12,41].

Ultrapure water produced using a Thermo Scientific GenPure UV/UF ultrapure water purification system (Thermo Fisher Scientific, Waltham, MA, USA) was used for reagent and treatment-solution preparation, irrigation of the corresponding water-control groups, and preparation of foliar spray solutions. Freshly produced ultrapure water had a resistivity of 18.2 MΩ·cm at 25 °C, an electrical conductivity (EC) of 0.056 μS cm^−1^, and a pH of 5.8 at 25 °C. For foliar application, the PMO stock suspension was diluted to the target concentrations with ultrapure water, and 0.05% (*v*/*v*) Silwet L-77 was added as a surfactant. All spray solutions were freshly prepared immediately before use. The PMO stock suspension was stored in sealed containers at 4 °C in the dark. The particle size, surface charge, crystal structure, elemental valence states, and ROS-scavenging properties described above were obtained from previously published characterizations of the PAA@Mn_3_O_4_ system by the supplying research group and were not independently remeasured for the specific batch used in the present study.

### 4.2. Experimental Design

#### 4.2.1. Pot Experiment

The experiment was conducted from March to April 2024 in the solar greenhouse of the Sugar Beet Physiology Research Institute, Inner Mongolia Agricultural University, Hohhot, Inner Mongolia Autonomous Region, China (111.710567° E, 40.812625° N). Seedling nursery pots (10 × 10 × 11 cm) were used, and the growing substrate was prepared by thoroughly mixing nutrient soil and vermiculite at a ratio of 3:1. Each pot contained approximately 0.59 kg of the growth substrate.

The experiment consisted of 14 actual treatment groups arranged in three independent experimental replicates. Within each experimental replicate, each treatment consisted of 10 individual pots, with one plant retained in each pot after thinning. Therefore, each experimental replicate contained 140 pots (14 treatments × 10 pots), resulting in a total of 420 pots across the three independent experimental replicates (14 treatments × 10 pots × 3 replicates).

The 14 actual treatments included one shared non-saline control (CK), one shared salt-stress control (SCK), four PAA@Mn_3_O_4_ nanoparticle treatments (PT1–PT4), four methyl jasmonate treatments (MT1–MT4), and four γ-aminobutyric acid treatments (GT1–GT4). In Table 1 and Figure 2, Figure 3, Figure 4, Figure 5, Figure 6, Figure 7, Figure 8, Figure 9, Figure 10, Figure 11, Figure 12 and Figure 13, PCK, MCK, and GCK represent alternative labels for the same non-saline control (CK), whereas SPCK, SMCK, and SGCK represent alternative labels for the same salt-stress control (SCK). These regulator-specific labels were used only to facilitate comparisons among the PAA@Mn_3_O_4_ nanoparticle, MeJA, and GABA concentration series and do not represent independent treatment groups or additional biological replicates.

After sowing, seedlings were thinned to one healthy plant per pot. Throughout the growth period, all pots were irrigated with equal volumes of water every 5 days. The controlled salt-shock treatment was initiated when the eighth true leaf of sugar beet seedlings was fully expanded. The salt treatment consisted of two successive irrigations with 300 mmol L^−1^ NaCl solution without gradual salt acclimation. During the first irrigation, 150 mL of NaCl solution was applied to each NaCl-treated pot at 9:00 a.m., whereas the corresponding non-saline control pots received the same volume of water at the same time. After the substrate was allowed to dry naturally following the first irrigation, the same treatment procedure was repeated. Thus, NaCl-treated and control pots followed the same irrigation schedule and received the same irrigation volume.

Four days after the second NaCl application, foliar application of the exogenous regulatory substances was performed. Each treatment received two foliar applications, with 75 mL of the corresponding treatment solution applied each time. All foliar spray solutions contained 0.05% (*v*/*v*) Silwet L-77 as a surfactant. The control treatments received the same volume of the corresponding control spray solution. Spraying was performed uniformly until leaf surfaces were completely wetted. The two foliar applications were conducted at 9:00 a.m. and 6:00 p.m. on the same day. The concentrations of PAA@Mn_3_O_4_ nanoparticles, MeJA, and GABA used in each treatment are shown in Table 1.

Sampling was performed seven days after foliar application. Functional leaves were collected from randomly selected plants within each experimental replicate. Samples collected from plants within the same experimental replicate were treated as subsamples rather than independent biological replicates. The three independent experimental replicates were used as biological replicates for statistical analysis. The collected leaf samples were immediately frozen in liquid nitrogen and subsequently stored at −80 °C for physiological and biochemical analyses.

#### 4.2.2. Field Experiment

The field experiment was conducted in 2024 at the Bayannur Institute of Agricultural and Animal Husbandry Sciences, Inner Mongolia Autonomous Region, China, as a one-year, two-site preliminary field evaluation. Two experimental sites with contrasting soil conditions were selected and are hereafter referred to as the saline–alkali soil site and the non-saline–alkali soil site. These terms were used to distinguish the two field environments investigated in this study. Sugar beet seeds were uniformly sown at both sites on 12 May. The saline–alkali soil site was located at the institute’s core experimental base (107.288203° E, 40.798697° N), where the soil pH was 8.83 and the electrical conductivity (EC) was 2.34 mS cm^−1^. The non-saline–alkali soil site was located at the institute’s Yuanziqu Experimental Base (107.176749° E, 40.877645° N), where the soil pH was 7.74 and the electrical conductivity (EC) was 1.89 mS cm^−1^. Detailed information on soil nutrient properties at the two experimental sites is provided in Table 2.

The field experiment was established using a randomized block design with four foliar treatments, including water-sprayed control (CK), PAA@Mn_3_O_4_ nanoparticles (PMO), methyl jasmonate (MeJA), and γ-aminobutyric acid (GABA), with three independent plots per treatment at each site. The selected concentrations from the preceding controlled salt-shock screening experiment were applied under field conditions, including 100 mg L^−1^ PMO, 100 mg L^−1^ MeJA, and 1000 mg L^−1^ GABA. The experimental unit in the field trial was defined as an individual plot. Plants within each plot were treated as subsamples, and plot means were used for statistical analyses. This design allowed evaluation of treatment effects under contrasting soil environments while avoiding pseudoreplication caused by treating individual plants within the same plot as independent biological replicates.

Compared with the non-saline–alkali soil site, the saline–alkali soil site had a higher total salt content (1.52 g kg^−1^ vs. 0.77 g kg^−1^), while differences were also observed in total N, available P, available K, and organic matter between the two sites. Identical field management practices were applied at both experimental sites. Fertigation was conducted three times during the growing season, with an irrigation volume of 450 m^3^ ha^−1^ per application. The fertilization regime was based on soil test results: 600 kg ha^−1^ of compound fertilizer (N:P:K = 12:18:15) was applied as basal fertilizer, while 225 kg ha^−1^ of urea and 120 kg ha^−1^ of potassium sulfate were applied as topdressings through the fertigation system during the growing period.

At each experimental site, the experiment was arranged in a randomized block design with four treatments and three blocks. Each block contained one plot for each treatment, resulting in three independent plots per treatment at each site. The four treatments consisted of three exogenous regulatory substance treatments and one water-sprayed control (Table 2). Each experimental plot contained four plastic-mulched rows, each 5 m in length, with a row spacing of 50 cm and a plant spacing of 18 cm. Thus, each experimental site contained 12 plots (4 treatments × 3 blocks), resulting in a total of 24 plots across the two sites. The individual plot was considered the experimental unit in the field experiment. The concentrations of the exogenous regulatory substances were selected based on the results of the preceding pot experiment and are listed in Table 3. The pot experiment was used for concentration screening under acute NaCl-induced salt stress, whereas the field experiment was used to preliminarily evaluate the effects of the selected treatments under saline–alkali and non-saline–alkali soil conditions. Therefore, the pot and field experiments were considered complementary stress models rather than physiologically equivalent stress models. Because each soil condition was represented by only one field site, treatment effects were evaluated separately within each site, and the two sites were not considered independent replicates of soil condition.

All foliar treatments were applied at the eight-leaf stage of sugar beet. Each treatment received two equal foliar applications of the corresponding regulator solution, with 1 L applied per plot at each application. The control plots were sprayed with an equal volume of water. Spraying was continued until the leaf surfaces were fully wetted and droplets began to run off along the leaf margins. All spraying procedures were performed under clear and windless weather conditions. The two applications were conducted at 09:00 and 18:00 on the same day. Plant samples were collected 20 days after foliar application. Three sugar beet plants were randomly sampled from each plot, and their functional leaves and storage roots were collected for subsequent physiological and biochemical analyses. The three plants sampled within the same plot were considered subsamples rather than independent biological replicates. Measurements obtained from the three plants within each plot were averaged to generate a single plot-level value before statistical analysis. Accordingly, statistical analyses for each treatment at each site were based on three independent plots (n = 3).

On 10 October 2024, all sugar beet plants within each plot were harvested to determine storage root yield and quality-related parameters. Yield and quality measurements were summarized at the plot level, and the resulting plot-level values were used as independent observations for statistical analysis.

### 4.3. Measurement of Physiological and Agronomic Parameters

#### 4.3.1. Determination of Seedling Morphological Parameters

After NaCl treatment and foliar application of the exogenous substances, seedling morphological phenotypes, including leaf curling, wilting, and chlorosis, were visually observed and documented. Roots were gently rinsed with deionized water to remove adhering substrate particles, and visible root developmental abnormalities were recorded. Seedlings from each treatment were photographed for qualitative documentation.

For quantitative morphological measurements, three plants were randomly selected from different pots of each treatment within each independent experimental replicate. Thus, a total of nine plants were measured for each treatment across the three independent experimental replicates. The three plants sampled within the same experimental replicate were treated as subsamples rather than independent biological replicates. Measurements from these three plants were averaged to generate a single replicate-level value for each treatment. Accordingly, the statistical analysis was based on three independent experimental replicates per treatment (n = 3).

Leaf number: The total number of leaves on each sampled plant, including senescent and withered leaves, was counted.

Taproot length: After the roots were gently rinsed and fibrous roots were removed, taproot length was measured from the first leaf scar to the root tip using a ruler.

Taproot diameter: The maximum diameter of the taproot was measured at its widest point using a ruler.

#### 4.3.2. Determination of Plant Fresh and Dry Weight

For fresh- and dry-weight measurements, three plants were randomly selected from different pots of each treatment within each independent experimental replicate. The three plants sampled within the same experimental replicate were treated as subsamples rather than independent biological replicates. Measurements obtained from these three plants were averaged to generate a single replicate-level value for each treatment. Accordingly, statistical analysis was based on three independent experimental replicates per treatment (n = 3).

Whole plants were gently rinsed with deionized water to remove adhering substrate particles and were then blotted with filter paper to remove surface moisture. Each plant was separated into shoot and root fractions using a scalpel, and the fresh weight (FW, g) of each fraction was measured using an analytical balance.

After fresh-weight determination, the samples were pre-dried in a cool, well-ventilated area to remove excess surface moisture. They were then placed in kraft paper bags, heated at 105 °C for 15 min in a forced-air drying oven, and subsequently dried at 80 °C to constant weight. After cooling to room temperature in a desiccator, the dry weight (DW, g) of the shoot and root fractions was measured separately.

For each independent experimental replicate, the fresh- and dry-weight values of the three sampled plants were averaged to obtain one replicate-level value for subsequent statistical analysis.

#### 4.3.3. Determination of Photosynthetic Physiological Parameters

All measurements were performed on the third or fourth fully expanded functional leaf counted from the shoot apex. The biological replication used for statistical analysis followed the experimental-unit structure described above, with three independent replicate-level observations per treatment (n = 3).

SPAD value: Leaf SPAD values were measured using a SPAD-520 Plus chlorophyll meter (Konica Minolta, Osaka, Japan). Three readings were taken from different positions on each leaf while avoiding the main vein and leaf margins, and the mean of the three readings was used as the SPAD value for that leaf. SPAD values were used only as a relative index of leaf chlorophyll status.

Gas-exchange parameters: Net photosynthetic rate (Pn), stomatal conductance (Gs), intercellular CO_2_ concentration (Ci), and transpiration rate (Tr) were measured using an LI-6400 portable photosynthesis system (LI-COR, Lincoln, NE, USA) between 09:00 and 11:00 on sunny days. The built-in LED light source was set to a photosynthetic photon flux density (PPFD) of 1200 μmol m^−2^ s^−1^, and the reference CO_2_ concentration was maintained at 400 μmol mol^−1^. Detailed records of leaf temperature, chamber flow rate, relative humidity, vapor pressure deficit (VPD), stabilization time, and measurement order were not retained after completion of the experiment and therefore could not be reliably reconstructed retrospectively.

Chlorophyll fluorescence parameters: Leaves were dark-adapted for at least 30 min before chlorophyll fluorescence measurements using a PlantView 230F in vivo chlorophyll fluorescence imaging system (Guangzhou Biolight Biotechnology, Guangzhou, China). The fluorescence parameters reported in this study were obtained using this imaging system. Detailed acquisition settings, including actinic-light intensity, saturation-pulse settings, and other imaging parameters, were not retained after completion of the experiment and therefore could not be reliably reconstructed retrospectively.

#### 4.3.4. Determination of Physiological and Biochemical Parameters

##### Antioxidant Enzyme Activities and MDA Content

Fresh leaf and storage root tissues were ground to a fine powder in liquid nitrogen, and 0.1 g of the powder was accurately weighed using an analytical balance. The activities of superoxide dismutase (SOD, WST-8 method), peroxidase (POD, guaiacol method) and catalase (CAT, ammonium molybdate method), as well as malondialdehyde (MDA) content (thiobarbituric acid method), were determined using commercial assay kits (Suzhou Grace Biotechnology Co., Ltd., Suzhou, China). All procedures were performed strictly following the manufacturer’s protocols, with three technical replicates per sample.

##### Osmotic Adjustment Substance Contents

Fresh leaf tissues were ground to a fine powder in liquid nitrogen. For each sample, 0.1 g of powder was used for free proline and soluble sugar assays, and 0.2 g for betaine determination. Free proline and betaine contents were measured using assay kits from Suzhou Grace Biotechnology Co., Ltd. (Suzhou, China), and soluble sugar content was determined via the anthrone colorimetric method using a kit from Nanjing Jice Biotechnology Co., Ltd. (Nanjing, China). All assays were performed with three technical replicates per sample.

##### Endogenous Hormone Contents

Fresh functional leaf samples were rapidly ground into a homogeneous fine powder in liquid nitrogen. A 0.1 g aliquot of the powdered sample was accurately weighed, and endogenous hormones were extracted according to the procedures specified in the corresponding kit instructions. Indole-3-acetic acid (IAA), zeatin riboside (ZR), and gibberellic acid (GA_3_) were quantitatively determined using enzyme-linked immunosorbent assay (ELISA) kits purchased from Shanghai Enzyme-linked Biotechnology Co., Ltd. (Shanghai, China). The corresponding catalogue numbers were ML077231 for IAA, ML077225 for ZR, and ML103716 for GA_3_.

All procedures were performed strictly according to the manufacturers’ instructions. Standard curves were generated using a series of standards at different concentrations, and the concentrations of IAA, ZR, and GA_3_ in the samples were calculated from the corresponding standard curves.

For consistency with the original figure and table labels, the abbreviations CTK and GA were retained in the figures and tables.

#### 4.3.5. Determination of Storage-Root Yield and Quality-Related Parameters

At harvest, plants in the border rows of each plot were excluded to minimize edge effects, and all remaining sugar beet storage roots were weighed to determine storage-root yield. Storage-root trimming was performed with reference to the Chinese national standard GB/T 10496-2018 [51], with minor adaptations to local field conditions.

Fifteen storage roots were randomly selected from each plot, and juice was obtained by punch sampling. Soluble solids content (SSC, °Brix) was measured directly using a handheld digital refractometer (ATAGO Co., Ltd., Tokyo, Japan). Because refractometric °Brix represents soluble solids rather than a direct measurement of sucrose concentration, the calculated sugar-related variables were treated as estimates. Estimated storage-root sugar concentration and estimated sugar yield were calculated as follows:

Estimated storage-root sugar concentration (%) = SSC (°Brix) × 0.85

Estimated sugar yield (t ha^−1^) = Storage-root yield (t ha^−1^) × Estimated storage-root sugar concentration (%)/100

The refractometer-derived SSC values were therefore reported separately from the calculated estimated storage-root sugar concentration, and the calculated sugar yield was reported as estimated sugar yield rather than as directly measured sugar yield.

### 4.4. Data Analysis

All statistical analyses were performed using IBM SPSS Statistics version 26.0 (IBM Corp., Armonk, NY, USA). Data are presented as the mean ± standard deviation (SD), unless otherwise stated. Measurements obtained from subsamples within the same independent experimental replicate were averaged before statistical analysis. For assays involving technical replicates, technical measurements from the same sample were first averaged and were not treated as additional independent observations.

For the pot experiment, statistical analyses were based on three independent experimental replicates per treatment (n = 3). Measurements obtained from subsamples within the same experimental replicate were averaged to generate a single replicate-level value. The PAA@Mn_3_O_4_ nanoparticle, MeJA, and GABA concentration series were analyzed separately. Within each analysis, the shared non-saline control (CK) and salt-stress control (SCK) were each included only once and were not treated as additional independent treatments or replicates.

For the field experiment, the individual plot was considered the experimental unit. Plants sampled within the same plot were treated as subsamples, and their measurements were averaged to obtain a single plot-level value before statistical analysis. Statistical analyses were therefore based on three independent plots per treatment at each experimental site (n = 3 plots). Because soil condition was confounded with field site, data from the two sites were analyzed separately.

Physiological and biochemical variables measured in leaf and storage-root tissues were analyzed separately. Technical replicates were averaged before statistical analysis and did not increase the number of independent observations.

For the pot experiment, treatment effects within each exogenous-substance concentration series were evaluated using one-way analysis of variance (ANOVA). For the field experiment, treatment effects were evaluated separately at each site using ANOVA appropriate for the randomized block design. When the overall ANOVA F-test indicated a significant treatment effect (*p* < 0.05), Duncan’s multiple range test was used for subsequent multiple comparisons and mean separation at the 0.05 significance level.

The significance level for all statistical tests was set at *p* < 0.05. All figures were generated using OriginPro 2023b software.

## 5. Conclusions

Combining pot-based concentration screening with field evaluation under saline–alkali conditions, this study evaluated the effects of foliar PAA@Mn_3_O_4_ nanoparticles (PMO), methyl jasmonate (MeJA), and γ-aminobutyric acid (GABA) treatments on the growth, physiological characteristics, yield, and quality-related traits of sugar beet under salt-affected conditions. All three foliar treatments exhibited concentration-dependent responses, and 100 mg L^−1^ PMO, 100 mg L^−1^ MeJA, and 1000 mg L^−1^ GABA were selected for subsequent field evaluation.

At the selected concentrations, PMO, MeJA, and GABA treatments were associated with improved growth and physiological performance under the imposed NaCl treatment and with positive yield-related responses under saline–alkali field conditions. These responses were accompanied by changes in growth characteristics, photosynthetic performance, antioxidant-related traits, osmolyte accumulation, and selected growth-related hormone levels. However, the present study characterized physiological responses following PMO application and did not directly determine nanoparticle-specific mechanisms, including in planta ROS-scavenging activity, nanozyme activity, nanoparticle uptake, or translocation.

Under saline–alkali field conditions, all three selected treatments increased storage-root yield and estimated sugar yield, although estimated storage-root sugar concentration decreased relative to SCK. PMO treatment increased storage-root yield and estimated sugar yield by 13.59% and 10.77%, respectively, compared with SCK. These results indicate a trade-off between storage-root yield and estimated storage-root sugar concentration under the evaluated field conditions.

Overall, the findings provide preliminary evidence supporting further agronomic evaluation of foliar PMO, MeJA, and GABA under salt-affected production conditions. Further studies are required to clarify the physiological and molecular processes associated with these treatment responses and to evaluate nanoparticle fate, residue safety, environmental effects, and the feasibility of repeated and multi-year field applications before broader agricultural use is considered.

## Figures and Tables

**Table 1:** Types and spray concentrations of exogenous substances in the pot experiment.

Treatment	Three Exogenous Substances	Spraying Concentration
PCK	Water	0
SPCK	NaCl	300 mmol/L
PT1	PMO	50 mg/L
PT2	PMO	100 mg/L
PT3	PMO	200 mg/L
PT4	PMO	300 mg/L
MCK	Water	0
SMCK	NaCl	300 mmol/L
MT1	MeJA	20 mg/L
MT2	MeJA	70 mg/L
MT3	MeJA	100 mg/L
MT4	MeJA	180 mg/L
GCK	Water	0
SGCK	NaCl	300 mmol/L
GT1	GABA	200 mg/L
GT2	GABA	500 mg/L
GT3	GABA	1000 mg/L
GT4	GABA	2000 mg/L

PCK, MCK, and GCK are alternative labels for the same shared non-saline control (CK), whereas SPCK, SMCK, and SGCK are alternative labels for the same shared salt-stress control (SCK). These regulator-specific labels are used only to facilitate comparison.

**Table 2:** Soil nutrient information at the test site.

Experimental Site	Total N (g kg^−1^)	Available P (mg kg^−1^)	Available K (mg kg^−1^)	Organic Matter (g kg^−1^)	Total Salts (g kg^−1^)	pH
Bayannur Experimental Base of Institute of Agricultural and Animal Husbandry Sciences	0.83	20.2	141.2	14.6	1.52	8.83
Yuanzigu Experimental Site	0.89	36.48	166.8	15.1	0.77	7.74

**Table 3:** Types and spray concentrations of exogenous substances in the field experiment.

Treatment	Three Exogenous Substances	Spraying Concentration
CK	Water	0
PT2	PMO	100 mg/L
MT3	MeJA	100 mg/L
GT3	GABA	1000 mg/L

CK represents the water-sprayed control. PT2, MT3, and GT3 represent the PAA@Mn_3_O_4_ nanoparticle, MeJA, and GABA treatments, respectively. The application concentrations used in the field experiment were selected based on the results of the preceding pot experiment. PAA@Mn_3_O_4_ nanoparticles, poly(acrylic acid)-coated Mn_3_O_4_ nanoparticles; MeJA, methyl jasmonate; GABA, γ-aminobutyric acid.

## Data Availability

Dataset available on request from the authors. The raw data supporting the conclusions of this article will be made available by the authors on request.
